# Supplementary figures and images for: A remarkable new deep-sea nereidid (Annelida: Nereididae) with gills
Source: PLoS One. 2024 Mar 6;19(3):e0297961. doi: 10.1371/journal.pone.0297961 (PMC10917260; doi:10.1371/journal.pone.0297961)

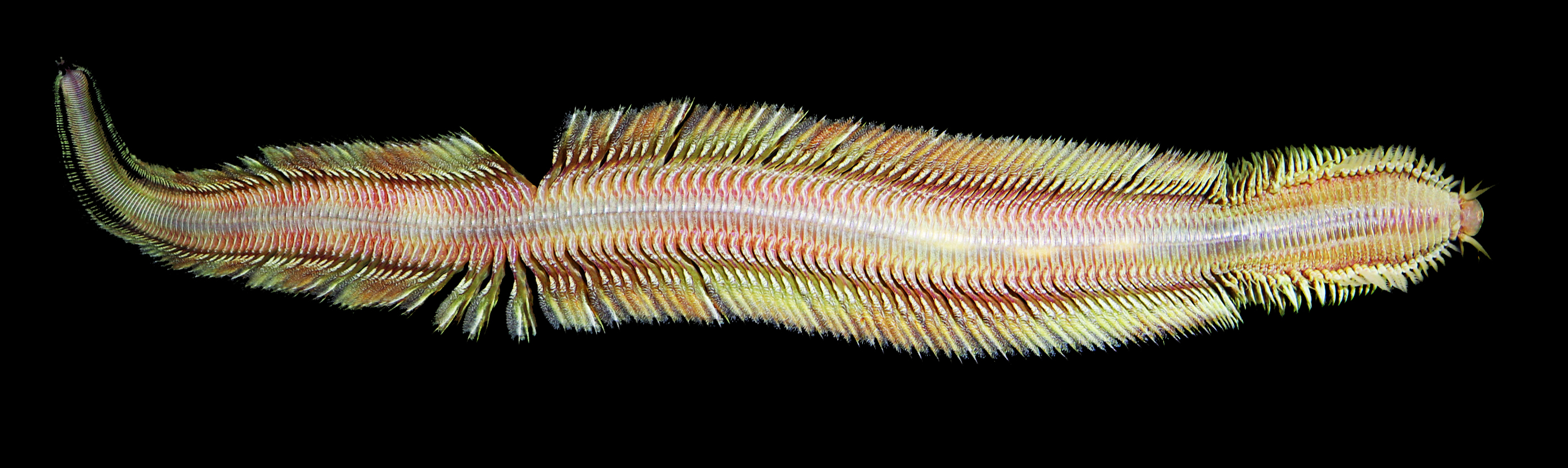

Supplement: S1 Fig — (JPG) [file pone.0297961.s001.jpg]
