# Supplementary material for: A remarkable new deep-sea nereidid (Annelida: Nereididae) with gills
Source: PLoS One. 2024 Mar 6;19(3):e0297961. doi: 10.1371/journal.pone.0297961 (PMC10917260; doi:10.1371/journal.pone.0297961)
Supplement: S1 Table — Data was sourced from Alves et al. 2023 [129]. New sequences are indicated in bold. Most sequence IDs from BOLD are marked with #. * Laeonereis cf. pandoensis (Monro, 1938) is used here instead of Laeonereis culveri (Webster, 1879) since the specimen was collected in Brazil. One terminal from Alves et al. 2023 [129] was not included here, Ceratonereis longiceratophora Hartmann-Schröder, 1985 as the there was no 16S sequence, the COI sequence (AY583701) is actually a flabelligerid and the 18S sequence appears to be of a hesionid. The correct spelling for sequences lodged on GenBank as Tylorrhynchus heterochaetus is Tylorrhynchus heterochetus. (DOCX) [file pone.0297961.s002.docx]

**S1 Table.** Accession numbers for the phylogenetic analysis of COI, 16S rRNA and 18S rRNA shown in Fig. Data was sourced from Alves et al. 2023 [131]. New sequences are indicated in **bold.** Most sequence IDs from BOLD are marked with #. * the name *Laeonereis* cf. *pandoensis* (Munro 1938) is used here instead of *Laeonereis culveri* (Webster 1879) since the specimen was collected in Brazil. One terminal from Alves et al. 2023 [131] was not included here, *Ceratonereis longiceratophora* Hartmann-Schröder, 1985 as the there was no 16S sequence, the COI sequence (AY583701) is actually a flabelligerid and the 18S sequence appears to be of a hesionid. The correct spelling for sequences lodged on GenBank as *Tylorrhynchus heterochaetus* is *Tylorrhynchus heterochetus.*

| **Nereididae ingroup** | COI | 16S rRNA | 18S rRNA |
| --- | --- | --- | --- |
| *Alitta succinea* (Leuckart, 1847) | MW825350 | MW826068 | MW826081 |
| *Ceratocephale abyssorum* (Hartman & Fauchald, 1971) | GQ426683 | GQ426618 | GQ426585 |
| *Ceratocephale loveni* Malmgren, 1867 |  | DQ442614 | DQ442616 |
| *Ceratonereis longiceratophora* Hartmann-Schröder, 1985 | AY583701 |  | AB106251 |
| *Ceratonereis* sp. | LIPOL031-08 # |  |  |
| *Cheilonereis cyclurus* (Harrington, 1897) | MF538532 | MF538532 |  |
| *Gymnonereis crosslandi* (Monro, 1933) | DISA414-18 # |  |  |
| *Gymnonereis* sp. | KY805815 | KY704332 |  |
| *Hediste atoka* Sato & Nakashima, 2003 | LC323006 | LC323043 | LC323072 |
| *Hediste diadroma* Sato & Nakashima, 2003 | KX499500 | KX499500 |  |
| *Laeonereis* cf. *pandoensis* (Munro 1938)* | MW825351 | MW826069 | MW826082 |
| *Namalycastis* *abiuma* (Grube, 1872) | KU351089 | KU351089 |  |
| *Namalycastis* *indica* (Southern, 1921) | MG759522 | MG759523 |  |
| *Namalycastis* jiaya Magesh et al., 2012 | HQ456363 | HM138706 | JX483867 |
| *Namanereis* *hummelincki* (Augener, 1933) | KT235957 |  |  |
| *Neanthes* *ceciliae* Steiner & Santos, 2004 | MW825352 | MW826070 | MW826083 |
| *Neanthes* *glandicincta* (Southern, 1921) | KY094478 | KY094478 |  |
| *Neanthes goodayi* | MZ407917 | MZ408676 |  |
| *Neanthes* *meggitti* (Monro, 1931) | MF958994 | MF959006 |  |
| *Nectoneanthes* *oxypoda* (Marenzeller, 1879) | HZPLY588- 13 # |  |  |
| ***Nectoneanthes oxypoda*** | **OL782599** | **OL782599** | **OR437940** |
| *Nereis anoculepitoka* | OR016053 | OR019766 |  |
| *Nereis mariellae* | OR016057 | OR019774 |  |
| *Nereis pelagica* Linnaeus, 1758 |  | AY340470 | AY340438 |
| ***Nereis pelagica*** | **OL782598** | **OL782598** | **OR437939** |
| *Nereis saramagoi* | OR016052 | OR019765 |  |
| *Nereis shinkai* | LC331618 | OR004585 |  |
| *Nereis* sp. | MF960765 | MF960765 |  |
| *Nicon maculata* Kinberg, 1865 | MW825353 | MW826071 |  |
| *Paraleonnates uschakovi* Chlebovitsch & Wu, 1962 | KX462988 | KX462988 |  |
| ***Pectinereis strickrotti* gen. nov., sp. nov.** | **OL782600** | **OL782600** | **OR437941** |
| *Perinereis aibuhitensis* (Grube, 1878) | KF611806 | KF611806 |  |
| *Perinereis anderssoni* Kinberg, 1866 | MW825354 | MW826072 | MW826084 |
| *Perinereis cultrifera (Grube, 1840)* | MN812983 | MN812983 | OQ732688 |
| *Perinereis nuntia* (Lamarck, 1818) | JX644015 | JX644015 |  |
| *Perinereis* sp. | MN823962 | MN823971 | OQ732689 |
| *Platynereis australis* (Schmarda, 1861) | MN830367 | MN830367 | OQ732690 |
| *Platynereis bicanaliculata* (Baird, 1863) | MN812984 | MN812984 | OQ732691 |
| *Platynereis dumerilii* (Audouin & Milne Edwards, 1833) | AF178678 | AF178678 |  |
| *Platynereis massiliensis* (Moquin- Tandon, 1869) | MN812985 | MN812985 | OQ732692 |
| *Platynereis* sp. 1 | MN830365 | MN830365 | OQ732693 |
| *Platynereis* sp. 2 | MW825355 | MW826073 | MW826085 |
| *Pseudonereis palpata* (Treadwell, 1923) | MW825356 | MW826074 | MW826086 |
| *Pseudonereis variegata* (Grube, 1857) | MN855134 | MN855213 | OQ732694 |
| *Simplisetia* cf. *erythraensis* (Fauvel, 1918) | EU835670 |  |  |
| *Tambalagamia fauveli* Pillai, 1961 | HZPLY601-13 # |  |  |
| *Tylorrhynchus heterochaetus* (sic) (Quatrefages, 1866) | KM111507 | KM111507 |  |
| **Outgroups** |  |  |  |
| *Arichlidon gathofi* Watson Rusell,2000 | MN855127 |  | OQ732695 |
| *Oxydromus pugettensis* (Johnson, 1901) | MN855132 | MN855211 | OQ732696 |
