# Supplementary material for: A remarkable new deep-sea nereidid (Annelida: Nereididae) with gills
Source: PLoS One. 2024 Mar 6;19(3):e0297961. doi: 10.1371/journal.pone.0297961 (PMC10917260; doi:10.1371/journal.pone.0297961)
Supplement: Attachment — Submitted filename: PONE-D-23-29948_REV.pdf [file pone.0297961.s006.pdf]

PLOS ONE

A remarkable new deep-sea nereidid (Annelida: Nereididae) with branchiae

--Manuscript Draft--

Manuscript Number:	PONE-D-23-29948
Article Type:	Research Article
Full Title:	A remarkable new deep-sea nereidid (Annelida: Nereididae) with branchiae
Short Title:	A new deep-sea branchiate nereidid
Corresponding Author:	Greg Rouse<br><br>UNITED STATES
Keywords:	polychaete; deep sea
Abstract:	Nereidid polychaetes are well known from shallow marine habitats, but their diversity in the deep sea is poorly known. Here we describe an unusual new nereidid species found at methane seeps off the Pacific coast of Costa Rica. Specimens of Pectinereis strickrotti gen. nov., sp. nov. had been observed dating back to 2009 swimming just above the seafloor at ~1,000 m depth but were not successfully captured until 2018. Male epitokes were collected as well as a fragment of an infaunal female found in a pushcore sample. The specimens were all confirmed as the same species based on mitochondrial COI. Phylogenetic analyses, including one based on available whole mitochondrial genomes for Nereididae, revealed no close relative, allowing for the placement of the new species in its own genus. This was supported by the unusual morphology, including pectinate branchiferous parapodial cirrostyles, hooked aciculae, elfin-shoe shaped ventral cirrophores, and elongate, fusiform dorsal ligules emerging basally to expanded cirrophores. Additionally, the morphology and phylogenetic relationships supported Dendronereidinae as monophyletic and is here delimited.
Order of Authors:	Tulio F. Villalobos-Guerrero
	Huč Sonja
	Tilic Ekin
	Avery Hiley
	Greg Rouse
Additional Information:	
**Question**	**Response**
**Financial Disclosure**<br><br>Enter a financial disclosure statement that describes the sources of funding for the work included in this submission. Review the submission guidelines for detailed requirements. View published research articles from *PLOS ONE* for specific examples.<br><br>This statement is required for submission and **will appear in the published article** if the submission is accepted. Please make sure it is accurate.	GWR NSF-OCE 0939557 US National Science Foundation https://www.nsf.gov. The funders had no role in study design, data collection and analysis, decision to publish, or preparation of the manuscript.

Powered by Editorial Manager® and ProduXion Manager® from Aries Systems Corporation

**Unfunded studies**<br>Enter: *The author(s) received no specific funding for this work.*<br><br>**Funded studies**<br>Enter a statement with the following details:<br>• Initials of the authors who received each award<br>• Grant numbers awarded to each author<br>• The full name of each funder<br>• URL of each funder website<br>• Did the sponsors or funders play any role in the study design, data collection and analysis, decision to publish, or preparation of the manuscript?<br>• **NO** - Include this sentence at the end of your statement: *The funders had no role in study design, data collection and analysis, decision to publish, or preparation of the manuscript.*<br>• **YES** - Specify the role(s) played.<br><br>* typeset	
**Competing Interests**<br><br>Use the instructions below to enter a competing interest statement for this submission. On behalf of all authors, disclose any competing interests that could be perceived to bias this work—acknowledging all financial support and any other relevant financial or non-financial competing interests.<br><br>This statement is required for submission and **will appear in the published article** if the submission is accepted. Please make sure it is accurate and that any funding sources listed in your Funding Information later in the submission form are also declared in your Financial Disclosure statement.<br><br>View published research articles from *PLOS ONE* for specific examples.	The authors have declared that no competing interests exist.

Powered by Editorial Manager® and ProduXion Manager® from Aries Systems Corporation

**NO authors have competing interests**<br><br>Enter: *The authors have declared that no competing interests exist.*<br><br>**Authors with competing interests**<br><br>Enter competing interest details beginning with this statement:<br><br>*I have read the journal's policy and the authors of this manuscript have the following competing interests: [insert competing interests here]*<br><br>* typeset	
**Ethics Statement**<br><br>Enter an ethics statement for this submission. This statement is required if the study involved:<br><br>• Human participants<br>• Human specimens or tissue<br>• Vertebrate animals or cephalopods<br>• Vertebrate embryos or tissues<br>• Field research<br><br>Write "N/A" if the submission does not require an ethics statement.<br><br>General guidance is provided below. Consult the submission guidelines for detailed instructions. **Make sure that all information entered here is included in the Methods section of the manuscript.**	Instituto Costarricense de Pesca y Acuicultura (permit INCOPESCA-CPI-003-12-2018) and Comisión Nacional para la Gestión de la Biodiversidad (permit R-070-2018- OT-CONAGEBIO)

**Format for specific study types**<br><br>**Human Subject Research (involving human participants and/or tissue)**<br>• Give the name of the institutional review board or ethics committee that approved the study<br>• Include the approval number and/or a statement indicating approval of this research<br>• Indicate the form of consent obtained (written/oral) or the reason that consent was not obtained (e.g. the data were analyzed anonymously)<br><br>**Animal Research (involving vertebrate animals, embryos or tissues)**<br>• Provide the name of the Institutional Animal Care and Use Committee (IACUC) or other relevant ethics board that reviewed the study protocol, and indicate whether they approved this research or granted a formal waiver of ethical approval<br>• Include an approval number if one was obtained<br>• If the study involved *non-human primates*, add *additional details* about animal welfare and steps taken to ameliorate suffering<br>• If anesthesia, euthanasia, or any kind of animal sacrifice is part of the study, include briefly which substances and/or methods were applied<br><br>**Field Research**<br><br>Include the following details if this study involves the collection of plant, animal, or other materials from a natural setting:<br>• Field permit number<br>• Name of the institution or relevant body that granted permission	
**Data Availability**<br><br>Authors are required to make all data underlying the findings described fully available, without restriction, and from the time of publication. PLOS allows rare exceptions to address legal and ethical concerns. See the PLOS Data Policy and FAQ for detailed information.	Yes - all data are fully available without restriction

A Data Availability Statement describing where the data can be found is required at submission. Your answers to this question constitute the Data Availability Statement and **will be published in the article**, if accepted.<br><br>**Important:** Stating 'data available on request from the author' is not sufficient. If your data are only available upon request, select 'No' for the first question and explain your exceptional situation in the text box.<br><br>Do the authors confirm that all data underlying the findings described in their manuscript are fully available without restriction?	
**Describe where the data may be found in full sentences. If you are copying our sample text, replace any instances of XXX with the appropriate details.**<br><br>• If the data are **held or will be held in a public repository**, include URLs, accession numbers or DOIs. If this information will only be available after acceptance, indicate this by ticking the box below. For example: *All XXX files are available from the XXX database (accession number(s) XXX, XXX.).*<br>• If the data are all contained **within the manuscript and/or Supporting Information files**, enter the following: *All relevant data are within the manuscript and its Supporting Information files.*<br>• If neither of these applies but you are able to provide **details of access elsewhere**, with or without limitations, please do so. For example:<br><br>*Data cannot be shared publicly because of [XXX]. Data are available from the XXX Institutional Data Access / Ethics Committee (contact via XXX) for researchers who meet the criteria for access to confidential data.*<br><br>*The data underlying the results presented in the study are available from (include the name of the third party*	All relevant data are within the manuscript and its Supporting Information files.<br>Sequences will be released on GenBank on publication.

and contact information or URL).<br>• This text is appropriate if the data are owned by a third party and authors do not have permission to share the data.<br><br>* typeset	
Additional data availability information:	

Powered by Editorial Manager® and ProduXion Manager® from Aries Systems Corporation

Manuscript

Click here to access/download;Manuscript;PectinereisFinal.docx

A new deep-sea branchiate nereidid

A Remarkable New Deep-Sea Nereidid (Annelida: Nereididae) with Branchiae

Tulio F. Villalobos-Guerrero[1], Sonja Huč [2], Ekin Tilic[2,3], Avery S. Hiley[2], Greg W. Rouse[2*]

[1] Department of Marine Ecology, Centro de Investigación Científica y de Educación Superior de Ensenada, Ensenada, Baja California, Mexico

[2] Scripps Institution of Oceanography, University of California San Diego, La Jolla, California, United States of America

[3] Department of Marine Zoology, Senckenberg Research Institute and Natural History Museum, Frankfurt am Main, Germany

*Corresponding author:

E-mail: grouse@ucsd.edu (GWR)

Abstract

Nereidid polychaetes are well known from shallow marine habitats, but their diversity in the deep sea is poorly known. Here we describe an unusual new nereidid species found at methane seeps off the Pacific coast of Costa Rica. Specimens of *Pectinereis strickrotti* gen. nov., sp. nov. had been observed dating back to 2009 swimming just above the seafloor at ~1,000 m depth but were not successfully captured until 2018. Male epitokes were collected as well as a fragment of an infaunal female found in a pushcore sample. The specimens were all confirmed as the same species based on mitochondrial COI. Phylogenetic analyses, including one based on available whole mitochondrial genomes for Nereididae, revealed no close relative, allowing for the placement of the new species in its own genus. This was supported by the unusual morphology, including pectinate branchiferous parapodial cirrostyles, hooked aciculae, elfin-shoe shaped ventral cirrophores, and elongate, fusiform dorsal ligules emerging basally to expanded cirrophores. Additionally, the morphology and phylogenetic relationships supported Dendronereidinae as monophyletic and is here delimited.

Introduction

The diversity of Nereididae de Blainville, 1818 compared to other polychaete clades of family rank is well documented, with over 700 accepted species [1–4]. Nereidids are generally known from coastal regions, commonly confined to shallow marine habitats, although they also occur in brackish, freshwater, and even moist terrestrial environments [1, 5–8]. However, ~10% of the total diversity is known from deep-sea habitats. Currently, 69 nereidid species found in 13 genera are known from below 500 m depth [9] from various habitats, including hydrothermal vents, cold seeps, polymetallic nodules, foraminifera ooze, whale carcasses, and sunken wood [10–15]. The best-represented genera are the polyphyletic *Neanthes* Kinberg, 1865 (16 species) and *Nereis* Linnaeus, 1758 (19 species), which includes the deepest known nereidid recorded, *Nereis profundi* Kirkegaard, 1956 from 7,290 m depth. One genus, the monotypic *Typhlonereis* Hansen, 1879 is exclusive to deep environments.

The deep sea encompasses a diverse, vast mosaic of understudied and poorly sampled habitats [16]. Several surveys that have sampled deep waters from distinct regions suggest polychaete biodiversity is severely underestimated [17–19], rendering our knowledge on it limited [20–22]. We can assume, therefore, that oceanic depths still host a vast number of yet undescribed nereidid species, although it is noteworthy that there is a relatively continual discovery of new deep-sea nereidids since about the 1960s (e.g., [6, 10–12, 14, 15, 23–36]).

While deep-water nereidids continue to be discovered, information about their behavior and inference about morphological adaptations has been scarce. According to Fauchald [11, 12], deep-sea nereidids share a few unusual prostomial and parapodial features when compared to shallow-living relatives, such as the absence of eyes, prolonged appendages and chaetae, and posterior chaetigers with extended notopodia and elongated neuropodia. Notably, the reduction of eyes and the elongation of parapodial appendages and chaetae fall under the general specialization to aphotic environments of some subterranean

and cave (troglobiotic) nereidids placed in Namanereidinae Hartman, 1959 [37–39]. These morphological adaptations inherent to the 'darkness syndrome' [40] have been shown for a few other polychaete clades containing cave and deep-sea members (e.g., Scalibregmatidae Malmgren, 1867; [41] and Aphroditiformia Levinsen, 1883 [42–45]), suggesting that these are also convergent evolutionary traits for nereidids living at aphotic depths.

Genetic tools have been instrumental in developing a better understanding of the diversity among nereidids. Several new species or previously-synonymized ones have been described, reinstated, or delimited through the integration of morphological data and molecular markers of apparently cryptic species (*e.g.*, [46–57]). Molecular data has also been utilized to investigate phylogenetic relationships at a broader scale among nereidid taxa [15, 57, 58]. Recently mitochondrial genomes (mitogenomes) have been sequenced for a variety of nereidid species (e.g., [59–66]), providing a rich dataset to assess phylogenetic relationships with more confidence. Alves et al. [67] tested the monophyly and phylogenetic relationships of the presently accepted subfamilies and provided an ancestral state reconstruction of pharyngeal structures using mitogenomic data. They rejected the monophyly of the subfamilies Nereidinae de Blainville, 1818 and Gymnonereidinae Banse, 1977 as currently recognized and revealed that the occurrence of papillae and paragnaths may not be reliable features to diagnose major groups.

This study describes a new, morphologically unusual, nereidid species belonging to a new genus using specimens found near methane seeps at ~1,000 m depth off Costa Rica in the eastern Pacific. This new species is remarkable for its dorsal and ventral anterior parapodial cirri that are modified as branchiae, and hooked-shaped posterior aciculae, both unique features among Nereididae. Its mitochondrial genome was sequenced as well as those for two shallow-water nereidid species. This allowed for a new mitogenomic analysis to evaluate the position and relationships of the new genus within Nereididae.

Material and methods

Specimen collection and preparation

Nereidid specimens were repeatedly seen swimming just above the seafloor at ~1,000 m depth off Costa Rica (Eastern Tropical Pacific) on cruises dating back to 2009 (Fig 1, S1 Video). However, it was not until 2018 that three epitokous males and one fragmented infaunal female were successfully collected near methane seeps of Mound 12 via the submersible DSV (deep submergence vehicle) *Alvin*, operated from the RV *Atlantis* (Fig 1B-D; S1 Video). The epitokes were suction sampled while swimming just above the bottom. The female fragment was incidentally cored with a PVC sediment push core. One specimen was also observed on a cruise in 2019 to the same region by the remotely operated vehicle (ROV) *SuBastian* operated from the RV *Falkor* (Fig 1E; S2 Video). See Levin *et al.* [13, 68] for details about location sites. The type specimens are deposited at the Benthic Invertebrate Collection, Scripps Institution of Oceanography, La Jolla, California, USA (SIO-BIC) and the Museo de Zoología (Universidad de Costa Rica), San José, Costa Rica (MZ-UCR).

Figure 1. *Pectinereis strickrotti* gen. nov., sp. nov. in life A, B, D. Several epitokous males swimming near methane seeps of Mound 12 (~1,000 m depth) of the Costa Rica margin and videoed via the submersible DSV *Alvin*. A. A frame grab from video taken on *Alvin* dive 4503 on Feb. 4, 2009. B and D. Frame grabs from video taken on *Alvin* dive 4987 on Nov. 2, 2018. C. A fragment of an atokous infaunal female collected at the same depth and locality via sediment pushcore on *Alvin* dive 4984 on Oct. 30, 2018. A white egg ~350 µm in diameter is visible on the exterior. Scalebar 1 mm. E. An epitokous male swimming near methane seeps of Parrita Scar (~1,000 m depth) of the Costa Rica margin. The specimen was initially caught via slurp with the ROV *SuBastian* (dive S0218, Jan. 11, 2019) but escaped. Images A, B, D, courtesy of Woods Hole Oceanographic Institute. E, courtesy of Schmidt Ocean Institute.

DNA extraction, sequencing, and genome skimming

DNA from each of the available specimens of the new species, as well as representatives of *Nectoneanthes oxypoda* (von Marenzeller, 1879) (SIO-BIC A13109) and *Nereis pelagica* Linnaeus, 1758 (SIO-BIC A6054) was extracted using the Zymo Research Quick-DNA™ Miniprep Plus kit, following the protocol supplied by the manufacturer. Mitochondrial cytochrome c oxidase subunit I (*COI*) sequences were obtained from the specimens via Sanger sequencing, with PCR amplification carried out using a mixture of 12.5 µl Apex 2.0x Taq Red DNA Polymerase Master Mix (Genesee Scientific, San Diego, California, USA), 1 µl each of the primers LCO1490/HCO2198 [69] (10 µM), 8.5 µl of ddH2O, and 2 µl of eluted DNA. A Mastercycler 5345 epGradient thermal cycler (Eppendorf, Hamburg, Germany) was used with the reaction protocol 94°C/180s – (94°C/30s – 47°C/45s – 72°C/60s) * 5 cycles – (94°C/30s – 52°C/45s – 72°C/60s) * 30 cycles – 72°C/300s. Final PCR products were purified with the ExoSAP-IT protocol (USB Affymetrix, Ohio, USA), and sequencing was performed by Eurofins Genomics (Louisville, Kentucky, USA). Sequences were assembled using Geneious v. 2022.2.2 (©Biomatters Ltd.; http://www.geneious.com/, New Zealand), and the new sequences were deposited to GenBank (OQ415952-OQ415955).

Genome skimming was conducted on the holotype of the new species, *Nectoneanthes oxypoda*, and *Nereis pelagica*. Prior to genome skimming, total DNA concentration for gDNA extractions was estimated using the Qubit dsDNA BR Assay Kit with a Qubit fluorometer (Invitrogen), and DNA quality was assessed with agarose gel electrophoresis at 100 V for 65 min. The DNA extractions were sent to Novogene (en.novogene.com) for library preparation and whole genome sequencing using 150 base pair (bp) paired-end reads on the Illumina NovaSeq 6000 platform (Illumina, San Diego, CA), resulting in 9.8-13.3 million paired-end raw reads per sample.

Mitochondrial genome assembly and annotation

Sequence reads were trimmed and cleaned with Trimmomatic v. 0.39 [70] before assembly with MitoFinder v. 1.4 [71] with The Invertebrate Mitochondrial Code (NCBI; transl_table = 5) used to translate the 13 protein-coding genes (PCGs). Complete records for all RefSeq annelid mitogenomes available on NCBI GenBank were used as the MitoFinder reference file, with MEGAHIT v. 1.2.9 [72] metagenomic assembler and Arwen v.1.2.3 [73] tRNA gene annotator selected for the assembly parameters. Resulting mitochondrial genes recovered in MitoFinder [71] contigs were checked for contamination using NCBI's Nucleotide BLAST. The MITOS Web Server [74] was used for mitogenome annotation. Geneious Prime v. 2022.2.2 [75] was used to manually finalize annotations, extract the 13 PCGs and two ribosomal RNA genes (rRNAs), and translate the PCGs into amino acids. Nuclear *18S* rRNA gene sequences (*18S*) were also mined out for the three species: using BBMap v. 38.87 [76], post-Trimmomatic paired-end reads were interleaved with the included reformat.sh script. Publicly available and closely related nereidid *18S* sequences were extracted in FASTA format from NCBI. Interleaved FASTQ files for each of the three species were mapped individually to the nereidid 18S sequences using Minimap2 v. 2.22 [77, 78], then SAMtools v. 1.13 [79] was used to extract the resulting mapped reads. The Map to Reference tool in Geneious Prime v. 2022.2.2 [75] was used to individually map the FASTQ mapped reads to the nereidid *18S* FASTA file for read coverage visualization and consensus sequence extraction. Newly assembled and annotated mitogenomes obtained in this study were deposited in GenBank with accession numbers OL782598-600 and the 18S sequences as OR437939-41 (Table 1; S1 Table).

Table 1. Mitochondrial sequences used for the phylogenetic analysis. Three new mitogenomes were generated for this study (**bold**). Also, four COI sequences generated for the holotype and three paratypes of *Pectinereis strickrotti* gen. nov., sp. nov. are listed. Note that the name *Laeonereis* cf. *pandoensis* (Munro, 1938) is used here instead of *Laeonereis culveri* (Webster, 1879) since the specimen was collected in Brazil. The correct spelling for sequences deposited in GenBank as *Tylorrhynchus heterochaetus* is actually *Tylorrhynchus heterochetus.*

**Taxon**	**Citation/Voucher**	**Collection Site**	**GenBank Accession Numbers**
Alitta succinea (Leuckart, 1847)	Alves et al. (2020)	USA: Florida	MN812981
Arichlidon gathofi Watson Russell, 2000 1	Alves et al. (2020)	USA: North Carolina	MN855126 (COI), MN855135 (COX2), MN855144 (COX3), MN855116 (CYTB), MN855107 (ATP6), MN855196 (ND5), MN855188 (ND4L), MN855178 (ND4), MN855154 (ND1), MN855171 (ND3), MN855164 (ND2)
Arichlidon gathofi Watson Russell, 2000 2	Alves et al. (2020)	Panama: Bocas del Toro	MN855127 (COI), MN855136 (COX2), MN855145 (COX3), MN855205 (ND6), MN855117 (CYTB), MN855108 (ATP6), MN855197 (ND5), MN855179 (ND4), MN855155 (ND1), MN855172 (ND3), MN855165 (ND2)
Bhawania goodei Webster, 1884	Alves et al. (2020)	Panama: Bocas del Toro	MN855128 (COI), MN855146 (COX3), MN855118 (CYTB), MN855198 (ND5), MN855189 (ND4L), MN855180 (ND4), MN855208 (16S), MN855156 (ND1), MN855173 (ND3), MN855166 (ND2)
Cheilonereis cyclurus (Harrington, 1897)	Park et al. (2017)	South Korea: Gangwondo	MF538532
Dendronereis chipolini Hsueh, 2019	Zhen et al. (2022)	China: Beibu Gulf	MW532084
Hediste diadroma Sato & Nakashima, 2003	Kim et al. (2016)	South Korea: Masan Port	KX499500
Hediste japonica (Izuka, 1908)	Park et al. (2020)	South Korea: Incheon	MN876864
Hesionides sp.	Alves et al. (2020)	Panama: Bocas del Toro	MN855129 (COI), MN855137 (COX2), MN855147 (COX3), MN855119 (CYTB), MN855109 (ATP6), MN855199 (ND5), MN855190 (ND4L), MN855181 (ND4), MN855157 (ND1), MN855174 (ND3), MN855167 (ND2)
Laeonereis cf. *pandoensis* (Munro, 1938)	Seixas et al. (2016)	Brazil: Rio de Janeiro	KU992689
Microphthalmus listensis Westheide, 1967 1	Alves et al. (2020)	Germany: Sylt	MN855130 (COI), MN855138 (COX2), MN855148 (COX3), MN855206 (ND6), MN855120 (CYTB), MN855110 (ATP6), MN855182 (ND4), MN855209 (16S), MN855158 (ND1)
Microphthalmus listensis Westheide, 1967 2	Alves et al. (2020)	Germany: Sylt	MN855139 (COX2), MN855149 (COX3), MN855121 (CYTB), MN855111 (ATP6), MN855200 (ND5), MN855191 (ND4L), MN855183 (ND4), MN855210 (16S), MN855159 (ND1), MN855175 (ND3)
Microphthalmus similis Bobretzky, 1870	Alves et al. (2020)	Germany: Sylt	MN855131 (COI), MN855140 (COX2), MN855150 (COX3), MN855122 (CYTB), MN855112 (ATP6), MN855201 (ND5), MN855192 (ND4L), MN855184 (ND4), MN855160 (ND1), MN855168 (ND2)
Namalycastis abiuma (Grube, 1872)	Lin et al. (2016)	China: Xiamen	KU351089
Neanthes glandicincta (Southern, 1921)	Lin et al. (2017)	China: Xiamen	KY094478
***Nectoneanthes oxypoda* (Marenzeller, 1879)**	**SIO-BIC A13109**	**Japan: Osaka Bay**	**OL782599**

***Nereis pelagica* Linnaeus, 1758**	**SIO-BIC A6054**	**Norway: Trondheim**	**OL782598**
Nereis sp.	Kim et al. (2017)	South Korea: Dok-do Isl.	MF960765
Nereis zonata Malmgren, 1867	Nam et al. (2021)	Beaufort Sea	MT980928
Oxydromus pugettensis (Johnson, 1901)	Alves et al. (2020)	USA: Washington	MN855132 (COI), MN855141 (COX2), MN855151 (COX3), MN855123 (CYTB), MN855113 (ATP6), MN855202 (ND5), MN855193 (ND4L), MN855184 (ND4), MN855211 (16S), MN855161 (ND1), MN855176 (ND3)
Oxydromus sp.	Alves et al. (2020)	Panama: Bocas del Toro	MN855133 (COI), MN855142 (COX2), MN855152 (COX3), MN855124 (CYTB), MN855114 (ATP6), MN855203 (ND5), MN855194 (ND4L), MN855186 (ND4), MN855212 (16S), MN855162 (ND1), MN855177 (ND3), MN855169 (ND2)
Paraleonnates uschakovi Chlebovitsch & Wu, 1962	Park et al. (2016)	South Korea: Ganghwa Isl.	KX462988
***Pectinereis strickrotti* gen. nov., sp. nov.**	**SIO-BIC A9836 (Holotype)**	**Costa Rica (Pacific): Mound 12**	**OL782600, (OQ415952-5 COI of holotype and the 3 paratypes)**
Perinereis aibuhitensis (Grube, 1878)	Kim et al. (2015)	South Korea: Ganghwa Isl.	KF611806
Perinereis cultrifera (Grube, 1840)	Alves et al. (2020)	France: Arcachon	MN812983
Perinereis nuntia (Lamarck, 1818)	Won et al. (2013)	South Korea: Yeosu	JX644015
Perinereis sp.	Alves et al. (2020)	Panama: Bocas del Toro	MN823962 (COI), MN823963 (COX2), MN823964 (COX3), MN823970 (ND6), MN823961 (CYTB), MN823960 (ATP6), MN823969 (ND5), MN823968 (ND4L), MN823967 (ND4), MN823972 (12S), MN823971 (16S), MN823965 (ND1), MN823966 (ND2)
Platynereis bicanaliculata (Baird, 1863)	Alves et al. (2020)	USA: Washington	MN812984
Platynereis cf. *australis*	Alves et al. (2020)	Chile: Chonchi	MN830367
Platynereis dumerilii (Audouin & Milne Edwards, 1833)	Boore & Brown (2000)	Europe	AF178678
Platynereis massiliensis (Moquin-Tandon, 1869)	Alves et al. (2020)	Wales: West Angle Bay	MN812985
Platynereis sp. 1	Alves et al. (2020)	Brazil: Ceara	MN830365
Platynereis sp. 2	Alves et al. (2020)	Brazil: Rio de Janeiro	MN830366
Pseudonereis variegata (Grube, 1857)	Alves et al. (2020)	South Africa: Western Cape	MN855134 (COI), MN855143 (COX2), MN855153 (COX3), MN855207 (ND6), MN855125 (CYTB), MN855115 (ATP6), MN855204 (ND5), MN855195 (ND4L), MN855187 (ND4), MN855214 (12S), MN855213 (16S), MN855163 (ND1), MN855170 (ND2)
Tylorrhynchus heterochaetus (sic) (Quatrefages, 1866)	Chen et al. (2016)	China: Nalong River	KM111507

Haplotype network and phylogenetic analyses

A haplotype network using *COI* data from the four specimens of the new species was generated with PopART v. 1.7 [80] using the TCS algorithm [81, 82]. A maximum likelihood (ML) phylogenetic analysis of concatenated *COI*, *16S rRNA*, and *18S rRNA* DNA sequences from across Nereididae (S1 Table) was analyzed, with each gene under the model GTR+FO+I+G4, chosen with ModelTest-NG v. 0.1.7 [83] and executed using raxmlGUI v. 2.0.10 [84]. The best ML tree was chosen after 100 ML runs (seed 581027) and support assessed via thorough bootstrapping (1,000 pseudoreplicates).

For the mitogenome-based phylogeny, alignments of the three newly generated mitochondrial genomes (two *rRNAs* and 13 PCGs translated into amino acids) along with relevant data on GenBank from several different studies (Table 1) were performed in Mesquite v. 3.61 [85] for each gene using the MUSCLE [86] algorithm with default settings. Data were partitioned by gene, with best-fit models for these partitions selected using ModelTest-NG v. 0.1.7. The substitution models selected were MTZOA for *ATP6*, *COI*, *COII*, *COIII*, *CYTB*, *ND1*, *ND3*, *ND4*, and *ND5*; MTMAM for *ND2*, *ND4L*, and *ND6*; MTREV for *ATP8*; TIM2 +I +G for *12S*; and TIM2 +G for *16S*. The data was then analyzed via an ML analysis with search and bootstrap parameters as with the three-gene analysis. Following Alves et al. [67], the terminals from Chrysopetalidae Ehlers, 1864 (*Arichlidon* Watson Russell, 1998 and *Bhawania* Schmarda, 1861), Microphthalmidae Hartmann-Schröder, 1971 (*Microphthalmus* Mecznikow, 1865 and *Hesionides* Friedrich, 1937), and Hesionidae Grube, 1850 (*Oxydromus* Grube, 1855) were used as outgroups.

Morphology

Methods for measurements of specimens (total body length, TL; length to chaetiger 15, L15; body width to chaetiger 15, W15), counting of structures and ridge patterns on the proboscis, and body dissections were explained elsewhere [87]. For practical purposes, decimal numbers

are used when measurements between two structures exceed one unit (*e.g.*, 1.3 times, 2.5 times, twice). In contrast, written fractions were used when those measurements were less than one unit (e.g., half, two-thirds, four-fifths).

Light microscopy observations were made using both stereo and compound microscopes. Specimens were photographed alive using a Canon EOS M5 camera. Preserved specimens were photographed with a Nikon D5100 or Canon Rebel T7 camera mounted on both the compound microscope and stereomicroscope. Some images were generated using stacks using Helicon Focus® 6 (Method C) or manually through Adobe Photoshop® CS6 (for chaetae). Parapodia of one specimen were processed for scanning electron microscopy (SEM) with a Zeiss Evo10 scanning electron microscope. Figure backgrounds were cleaned and darkened or lightened as necessary without manipulating the actual specimen. Parapodia were shown in anterior views unless otherwise stated.

Descriptions of the species are yielded on the holotype morphology unless otherwise stated. The methods performed, terminology, and standardized definitions established for overall nereidid features either newly proposed, partially readapted, or based upon references cited in Villalobos-Guerrero et al. [4] were followed. These authors proposed a division of the dorsal ligule into two main regions: proximal and distal. However, we consider that the proximal dorsal ligule ([4]: Fig 1C, dld) is instead the basal part of dorsal cirrus, namely the dorsal cirrophore, and the dorsal cirrus ([4]: Fig 1C, dc) is, therefore, the dorsal cirrostyle; whereas the distal region ([4]: Fig 1C, dlp) corresponds to the dorsal ligule itself. This is grounded on the circulatory system's arrangement in some nereidids' enlarged notopodia. The placement and irrigation of the notopodial vessels running alongside the lateral margins of such an enlarged structure are homologs. These have distinctly been illustrated in *A. succinea* Leuckart, 1847 (see [88]: 75, Fig 4G, J, as *Nereis limbata*), *Namalycastis abiuma* (Grube, 1872) (see [89]: 23, Fig 6, arrows in dorsal cirri) and *Stenoninereis* species (see [90]:

98, Fig 1B, solid red lines). This homology is also evident in several other Nereidinae, Gymnonereidinae, and Namanereidinae members, such as *A. acutifolia* (Ehlers, 1901) ([49]: 168, 3E, F), *Dendronereis aestuarina* Southern, 1921 ([91]: Pl. 20, Fig 4E, F), *Gymnonereis sibogae* (Horst, 1918) ([92]: 39, Fig 32b), *Namalycastis borealis* Glasby, 1999 ([38]: 33, Fig 5H), *Namanereis occulta* (Conde-Vela, 2013) ([38]: 33, Fig 5G), *Neanthes micromma* (Harper, 1979) ([93]: 100, Fig 6), and among others. All these taxa share the distal placement of the dorsal cirrostyle, which is easily recognized by the articulation and thickened basal tegument (sometimes barely evident in *Namalycastis*), and the reduced dorsal ligule when present on the fully expanded dorsal cirrophores. Nicoll [88] and Kaufmant [94] also found similar irrigation of the notopodial vessels in *A. virens* Sars, 1834 and *Hediste diversicolor* (Müller, 1776) (both as *Nereis*), respectively. However, the position of vessels and the innervations of capillaries were different, the dorsal cirrophore was only slightly enlarged, and the dorsal cirrostyle was sited sub-medially on the notopodia.

Finally, the relative extension of parapodial structures was described following Villalobos-Guerrero & Carrera-Parra [49]. However, the dorsal ligule and the parapodial cirrostyles were measured in comparison with the entire length of the parapodial cirrophore in natatory chaetigers of epitokous specimens. The first and last natatory chaetigers of epitokes were determined by the appearance/disappearance of additional parapodial lobes, particularly the expanded neuropodial postchaetal lobe.

Nomenclatural acts

The electronic edition of this article conforms to the requirements of the amended International Code of Zoological Nomenclature, and hence the new names contained herein are available under that Code from the electronic edition of this article. This published work and the nomenclatural acts it contains have been registered in ZooBank, the online registration system for the ICZN. The ZooBank LSIDs (Life Science Identifiers) can be

resolved, and the associated information viewed through any standard web browser by appending the LSID to the prefix “http://zoobank.org/”. The LSID for this publication is: urn:lsid:zoobank.org:pub:9E9C5CD6-EFE8-4B90-A63F-96A4544AC60B.

Results

Haplotype network and Phylogenetic analysis

The four *COI* sequences obtained from the new species were all unique, but varied by a maximum of less than 1%, five base pairs out of an alignment of 676 bases (Fig 2A). The fragment of an atokous infaunal female (Fig 1C) was clearly the same species as the epitokous males (Figs 1A, B, D) and differed by only 3-5 base pairs. The ML phylogenetic analysis (log likelihood= -37894.608366) based on the concatenated *COI*, *16S*, and *18S* DNA dataset of 4,320 bases (S1 Fig) showed the new taxon under study here (*Pectinereis strickrotti* gen. nov., sp. nov.) with no well-supported close relationships among the other Nereididae but was well nested within a strongly supported Nereidinae (S1 Fig). It did form a clade with a *Nicon* terminal, but with low support. *Paraleonnates* was the sister group to Nereidinae, though with moderate support. Gymnonereidinae was not monophyletic, with *Tylorrhynchus heterochetus* recovered as sister to all other Nereididae and the remaining gymnonereidins as sister group to *Dendronereis*.

The mitochondrial genome order for all the three newly sequenced taxa was the same as one of the two observed gene orders for Nereididae, identified as Group I by Alves et al. [67]. The newly generated mitogenomes of three nereidids, plus those from 23 other species (plus outgroups), resulted in a concatenated sequence alignment of 2,325 sites for the two *rRNA* genes and 3,829 amino acids for the 13 PCGs. The ML tree (log likelihood= -127521.3745) showed a high bootstrap support (>90%) for many clades (Fig 2B), though

some key nodes were recovered with lower support. Allowing for the additional terminals used here, the results were largely congruent with those of Alves et al. [67]. Two major clades were found: Clade I was a well-supported Nereidinae, while Clade II, with relatively low support, consisted of species belonging to Dendronereidinae, Gymnonereidinae, Namanereidinae, and *Paraleonnates* (Fig 2B). Dendronereidinae was represented by two terminals, *Dendronereis chipolini* Hsueh, 2019 and *Neanthes glandicincta* (Southern, 1921), although based on the shallow genetic distance between the individuals, the latter terminal is apparently a misidentification. The placement of *Pectinereis strickrotti* gen. nov., sp. nov. within Nereidinae was as the poorly supported sister group to a clade comprised of *Alitta*, *Hediste*, *Nectoneanthes*, *Perinereis*, *Platynereis*, and *Pseudonereis* terminals. The newly generated mitogenome for the type species of *Nereis*, *N. pelagica*, formed a well-supported clade with *Nereis zonata* Malmgren, 1867 while *Nectoneanthes oxypoda* was a well-supported sister group to *Alitta succinea* (Fig 2B).

Figure 2. Haplotype analysis and mitogenome phylogeny. A. Haplotype network of *COI* data acquired for the three male and one female specimen of *Pectinereis strickrotti* gen. nov., sp. nov. The holotype sequence (male epitoke) is marked with * and was five base pairs different from the female (infaunal fragment). B. Maximum likelihood (ML) tree derived from analysis of the concatenated 15-gene mitochondrial genome dataset, with the 13 PCGs translated to amino acids. Support values at nodes are bootstrap support percentages after 1,000 pseudoreplicates.

Taxonomy

Family NEREIDIDAE de Blainville, 1818

Subfamily NEREIDINAE de Blainville, 1818

Pectinereis *Villalobos-Guerrero, Huč, Tilic, Hiley & Rouse* **gen. nov.**

urn:lsid:zoobank.org:act:17ECB80D-BCC4-42F0-9F4E-3DD42EEF5EAA

Type species. *Pectinereis strickrotti* Villalobos-Guerrero, Huc, Tilic, Hiley & Rouse sp. nov.

Diagnosis

Prostomium with anterior region entire; longitudinal groove present. Paired antennae present. Palpophores sub-conical, transverse groove conspicuous; palpostyles digitiform. Eyes and lens absent. Anterior achaetous segmental region (tentacular belt) without ventrolateral projections, bearing four pairs of enlarged cirri. Maxillary and oral rings of proboscis cylindrical. Areas of proboscis with paragnaths only. Paragnaths conical only, evenly spaced, present on both maxillary and oral ring of proboscis. Ridge pattern of pharyngeal areas VI-V-VI: λ-shaped. Segmental glandular patches present. Notopodia well developed from third chaetiger. Body of epitokes divided into two regions: pre-natatory and natatory. Cirrostyles of parapodial cirri remarkably modified in first pre-natatory chaetigers, as comb-like branchiae with digitiform filaments, smooth in following chaetigers. Cirrostyles of dorsal cirri attached sub-distally to cirrophore in natatory chaetigers. Cirrophore of dorsal cirri markedly expanded with blunt, distal upper and lower lobes in natatory chaetigers; two divergent vessels running lengthwise. Cirrostyles of ventral cirri single. Cirrophore of ventral cirri markedly elongated, acuminated, with lower lamella only, elfin-shoe shaped in natatory chaetigers. Dorsal, median, and ventral ligules present. Dorsal ligule elongate, fusiform, emerging from mid-lower edge of dorsal cirrophore in natatory chaetigers. Notopodial prechaetal lobe present throughout body. Median and ventral ligule smooth, fusiform. Neuropodial postchaetal lobe present throughout body, markedly expanded in natatory chaetigers, with distal projection. Neuropodial superior lobe absent. Neuropodial inferior lobe present, restricted to a few anterior chaetigers. Aciculae mostly dark throughout. Notoaciculae absent in first two chaetigers; non-expanded basal end in natatory chaetigers. Notochaetae with homogomph spinigers. Neurochaetae of both fascicles with homogomph spinigers and homogomph falcigers. Sesquigomph epitoke spiniger ensiform, present in

notopodia and both fascicles of neuropodia. Blade of falcigers very long, distal end obliquely truncate, terminal tooth without loop.

Etymology

This genus is named by combining the Latin word *pectinis* (= 'comb') with the name of the type genus of the family, *Nereis*. The name emphasizes the comb-like parapodial cirri (branchiae) in the pre-natatory chaetigers formed by digitiform filaments. The gender is feminine, as the stem genus-group name.

Remarks

Branchiae in nereidids are vascularized parapodia structures exclusively present in two shallow-water and estuarine genera: *Dendronereis* Peters, 1854 and *Dendronereides* Southern, 1921. The branchiae are modified dorsal cirrophores with multiple filaments starting at least some chaetigers after the first one; however, in the deep-water *Pectinereis* gen. nov., the branchiae are modified dorsal cirrostyles of both dorsal and ventral cirri present from the first chaetiger to a few anterior ones. In addition, *Pectinereis* gen. nov. can readably be distinguished from *Dendronereis* and *Dendronereides* by having an anteriorly complete prostomium, two neuropodial (postchaetal and inferior) lobes, and paragnaths only on both pharyngeal rings, whereas those two genera have an anteriorly indented prostomium, at least three neuropodial lobes, and papillae only on both pharyngeal rings.

The cirrophore of ventral cirri is poorly developed compared to that in the dorsal cirri and is usually overlooked in the systematics of nereidids. However, even when it is enlarged with additional lamellae in some epitoke nereidids, it is not distinguished from the cirrostyle and is usually referred to as 'ventral cirrus.' *Pectinereis* gen. nov. is the single nereidid genus that shows a markedly elongated and acuminated ventral cirrophore with lower, drop-shaped lamellae only in natatory chaetigers, giving the appearance of elfin-shoe shaped ventral cirri;

whereas epitoke species from other genera present a short, cylindrical ventral cirrophore with lower, reniform lamella and at least one upper lamellae in the same chaetigers.

Finally, the dorsal ligule and the markedly enlarged dorsal cirrophore seen in *Pectinereis* gen. nov. are present in middle and posterior chaetigers of several non-branchiferous nereidid genera, whether or not they are in an epitoke stage; for instance, *Cheilonereis* Benham, 1916, *Nectoneanthes* Imajima, 1972, some species of *Alitta* Kinberg, 1865, *Neanthes* Kinberg, 1865, *Nereis* Linnaeus, 1758, *Paraleonnates* Khlebovich & Wu, 1962, *Perinereis* Kinberg, 1865, *Pseudonereis* Kinberg, 1865. Nonetheless, the new genus can be distinguished from them all because the elongate, fusiform dorsal ligule is attached basally to the expanded cirrophore of dorsal cirri in middle and posterior chaetigers, giving the appearance of a bifurcate cirrophore. In the '*A. succinea*' species complex and some species of *Neanthes, Nereis*, *Perinereis*, and *Pseudonereis,* the dorsal ligule is smaller and shifted toward the distal end of the expanded dorsal cirrophore in middle and posterior chaetigers and sometimes is completely reduced. In species of the '*A. virens*' species complex, *Cheilonereis, Nectoneanthes*, and some *Paraleonnates,* the dorsal ligule is broadly enlarged and embraces entirely the expanded dorsal cirrophore in the same chaetigers.

Pectinereis strickrotti *Villalobos-Guerrero, Huč, Tilic, Hiley & Rouse* **sp. nov.**

Figs 1, 3−7

urn:lsid:zoobank.org:act:E143D2DF-BDD4-4E61-B5E7-5218EDD6B2A5

Material examined

Holotype

SIO-BIC A9836, epitoke male, Mound 12, Costa Rica, Pacific Ocean (8.929° N; 84.313° W), 02 Nov. 2018, 1,001−1,010 m, dive AD4987 (black slurp), coll. E. Cordes, E. Cowell,

R/V *Atlantis*, DSV *Alvin*, fixed in 95% EtOH, swimming near the bottom, good condition. GenBank *COI* sequence OQ415952, mitochondrial genome OL782600, 18S OR437941.

Paratypes

One epitoke male (MZUCR XXXX, was SIO-BIC 9837), same data as holotype, good condition, GenBank *COI* sequence OQ415953; one epitoke male (SIO-BIC A9889), Mound 12, Costa Rica, 30 Oct. 2018, 997 m, dive AD4984 (red slurp), coll. S. Goffredi, O. Pereira, R/V *Atlantis*, DSV *Alvin*, fixed in 10% formalin, swimming near the bottom, good condition. GenBank *COI* sequence OQ415954; one incomplete female, mid-body only (SIO-BIC A9891), Mound 12, Costa Rica, 30 Oct. 2018, 996 m, dive AD4984, coll. S. Goffredi, O. Pereira, R/V *Atlantis*, DSV *Alvin*, sediment of pushcore, fixed in 10% formalin. GenBank *COI* sequence OQ415955.

Description, holotype epitoke male.

Incomplete, 69 mm LT, 13 mm L15, 5 mm W15, with 150 chaetigers (paratype SIO-BIC A9889 complete with 192 chaetigers). General body color yellowish to reddish with mid-dorsum of chaetigers gray in live specimens, without pigmentation patterns but marked iridescence throughout (Fig 3A, B); body cream (Fig 3C) with faint traces of brownish pigmentation in palpophore and palpostyles (Fig 4A), and single transverse row of same color on dorsum of preserved specimens, more enhanced on posterior chaetigers.

Figure 3. *Pectinereis strickrotti* gen. nov., sp. nov. male anatomy. A, B, D-F. Paratype (SIO-BIC A9889), epitokous male: A, whole body of living specimen in dorsal view; B, anterior region of living specimen in dorsal view; D, prostomium of preserved specimen in frontal view; E, posterior end of preserved specimen in dorsal view; F, post-natatory chaetigers and pygidium of preserved specimen in dorsolateral view. C. Holotype (SIO-BIC A9836), epitokous male: anterior region of preserved specimen in dorsal view. Scale bars: A, ~20 mm; B, ~5 mm; C, 5 mm; D, 1 mm; E, 3 mm; F, 0.5 mm. Credits: A, B, Ekin Tilic; C, Tulio Villalobos; D-F, Greg Rouse.

Figure 4. *Pectinereis strickrotti* gen. nov., sp. nov. male anatomy details A, B, D, E. Holotype (SIO-BIC A9836), epitokous male: A, prostomium and everted proboscis in dorsal

view (arrow pointing additional abnormal antennae); B, everted proboscis in ventral view; C, everted proboscis in lateral view; E, branchiferous chaetigers in ventrolateral view (numbers referring to chaetiger). D. Paratype (SIO-BIC A9889), epitokous male: dissected anterior end in ventral view. Abbreviations: brd, dorsal branchiae; brv, ventral branchiae; pb, pharyngeal bulb; oe, esophagus. Scale bars: A–C, 2 mm; D, 3 mm; E, 1 mm. Credits: A-C, E: Tulio Villalobos; D, Greg Rouse.

Prostomium pear-shaped (Figs 3C, D, 4A), bent downwards in paratypes with non-everted proboscis, division between regions barely seen; anterior region distally entire, sub-rounded, as long as posterior region; anterolateral gap between antenna and palpophore narrow, as wide as basal diameter of antennae (Fig 3D). Nuchal organs covered by achaetous anterior segmental region (tentacular belt) bearing enlarged cirri, deeply embedded, medium size, as wide as basal diameter of posterodorsal enlarged anterior cirri.

Palpophores sub-conical, slightly thick, as long as wide (Figs 3D, 4A, C), as long as prostomium, bent downwards in paratypes with non-everted proboscis; sub-distal transverse groove distinct (Fig 4A). Palpostyles oval, as wide as one-quarter of palpophore (Figs 4D, 4A). Antennae abnormally as two pairs (Figs 4A), one pair only in paratypes; tapered, conical, medium size, as long as one-half of prostomial posterior region; antennae slightly separated by gap as wide as one-third of basal diameter of antenna (Figs 3D, 4A). Eyes and lens absent (Figs 3D, 4A).

Anterior achaetous segmental region markedly contracted owing to fixation and protrusion of proboscis, with straight anterior margin, bearing enlarged anterior cirri. Enlarged anterior cirri with rugged, non-articulated cirrostyles (Fig 3C, D). Anterodorsal cirrostyles extending backwards to chaetiger 4 (3 in paratype). Anteroventral cirrostyles longer than palpophores, as long and thick as posteroventral cirrostyles. Posterodorsal cirrostyles longest, extending backwards to chaetiger 8 (9 in paratype). Posteroventral cirrostyles extended laterally over middle of prostomial posterior region. Dorsal and

posteroventral cirrophores cylindrical, anteroventral cirrophores ring-shaped; anteroventral cirrophores as wide as posteroventral cirrophores.

Proboscis everted, with maxillary and oral rings cylindrical, wider than long (Fig 4A–C). Proboscis structures observed in holotype only. Jaws slightly crenulate (Fig 4A, B), reddish in distal third, remaining amber; with faint traces of 5 short denticles; 2 canals emerging from pulp cavity.

Paragnaths present on both maxillary and oral rings of proboscis, all conical, dark red and brown in maxillary ring, brownish in oral ring (Fig 4A–C); plate-like basements absent. Area I: 18, five slightly regular rows of uneven cones in broad, triangular patch, medial row cones largest (Fig 4A, C). Areas IIa: 26, IIb: 29, two to three regular longitudinal rows of uneven cones in L-shaped patch, distal cones larger (Fig 4A, C). Area III: 42, five irregular rows of uneven cones in broad, rectangular patch; proximal and outer cones shorter; 9 and 11 laterally-isolated cones in three slightly regular longitudinal rows (Fig 4B, C). Areas IVa: 33 and IVb: 32, three slightly curved longitudinal rows of uneven cones in L-shaped patch, distal cones larger forming one regular, transverse row (Fig 4B, C). Area V: 0 (Fig 4A). Areas VIa: 5 and VIb: 5, one slightly regular, transverse row of even cones (Fig 4A). Areas VII–VIII: 47, two well-separated bands of even cones on ridges only (absent in furrows), as isolated irregular patches; anterior band consisting of one regular transverse row of six cones (two on area VII, one on each ventral ridge of area VIII); posterior band with two transverse irregular rows (4–5 on each ventral ridge) (Fig 4B, C). Ridges of areas VI–V–VI with λ-shaped pattern (Fig 4A). Gap between area VI and areas VII–VIII broad, as wide as distal end of palpophore. Paired esophageal caeca absent (Fig 4D).

Body divided into two distinct regions although lacking posterior end, complete paratypes with four regions (Fig 3A): Holotype consisting of pre-natatory region with 31 chaetigers (Fig 3C) and natatory region with 119 chaetigers, becoming gradually narrower

towards posterior end from about chaetiger 125; in paratypes, pre-natatory region with 30–31 chaetigers (Fig 3A, B), natatory region with 94 chaetigers (Fig 3A), post-natatory region with 54 chaetigers (Fig 3A, E), and pre-pygidial region with 13 chaetigers (Fig 3E, F).

Pre-natatory region (Fig 5A–E) with notopodia consisting of dorsal cirri with cirrostyle and cirrophore, dorsal ligule, notopodial prechaetal lobe, and median ligule in biramous parapodia; and neuropodia consisting of neuroacicular ligule with inferior and postchaetal lobes, ventral ligule, and ventral cirrus with cirrostyle and cirrophore (neuropodial superior lobe not developed). First 17–18 dorsal and 14 ventral cirrostyles markedly modified as pectinate branchiae (Figs 3B, C, 4E, 5A–D): dorsal branchiae thick, becoming narrower and extending beyond dorsal ligule in parapodia 1–13 (Fig 4E), reducing in size progressively to become as long as dorsal ligule in chaetigers 17–18; ventral branchiae narrower than dorsal ones, becoming slenderer and extending beyond ventral ligule in chaetigers 1–14 (Fig 4E). Dorsal and ventral branchia with filaments on upper edge (Figs 4E, 5A–D); dorsal branchial filaments becoming shorter and narrower toward distal end of branchia, 6–11 filaments, more abundant in parapodia 3–14; ventral branchial filaments becoming longer and thicker toward middle of branchia, 2–10 filaments, more abundant in parapodia 4–10 and decreasing drastically in following branchiated parapodia (Fig 6). Dorsal cirrostyles of parapodia 18–19 to 31 and ventral cirrostyles of parapodia 15–31 cirriform, smooth (Fig 5E); dorsal and ventral cirrostyles elongating gradually to become as long as dorsal ligule and ventral ligule, respectively. Dorsal and ventral cirrophores enlarging from chaetigers 28 and 19, respectively. Parapodial ligules long, slender, tapering; dorsal ligule sharper, subequal to median ligule (Fig 5A, B, E). Notopodial prechaetal lobe present from chaetiger 3, long, slender, tapering, as long as three-quarters or four-fifths of median ligule (Fig 5A, B, E). Neuropodial postchaetal lobe conical, slightly longer than neuroacicular ligule (Fig 5A). Inferior lobe slender, conical, longer than neuroacicular ligule in parapodia 1–4

(Fig 5A), blunt and subequal to neuroacicular ligule in following parapodia (Fig 5B, E). Neuroacicular ligule shorter than ventral ligule (Fig 5A, B, E).

Figure 5. *Pectinereis strickrotti* gen. nov., sp. nov. male parapodia. A-H. Holotype (SIO-BIC A9836), epitokous male: A, parapodium of chaetiger 1; B, parapodium of chaetiger 10; C, parapodial dorsal cirrus of chaetiger 5; D, parapodial ventral cirrus of chaetiger 5; E, parapodium of chaetiger 22; F, parapodium of chaetiger 33; G, parapodium of chaetiger 55; H, parapodium of chaetiger 146. I. Paratype (SIO-BIC A9889), epitokous male: parapodium of posterior most (PM) chaetigers. White arrows indicate branchial filament. Abbreviations: dl, dorsal ligule; dp, dorsal cirrophore; ds, dorsal cirrostyle; in, inferior lobe; ml, median ligule; po, neuropodial postchaetal lobe; pr, notopodial prechaetal lobe; vl, ventral ligule; vp, ventral cirrophore; vs, ventral cirrostyle. Scale bars: A, B, E-H, 0.5 mm; C, D, I, 0.2 mm. Credits: A-H, Tulio F. Villalobos; I, Greg Rouse.

Figure 6. Distribution of dorsal and ventral branchial filaments of right parapodia from the holotype (SIO-BIC A9836) of *Pectinereis strickrotti* gen. nov., sp. nov.

Natatory region (Fig 5F–H) with notopodia consisting of dorsal cirri with cirrostyle and bilobate cirrophore, dorsal ligule, notopodial prechaetal lobe, and median ligule; neuropodia consisting of neuroacicular ligule with inferior and postchaetal lobes, ventral ligule, ventral cirri with cirrostyle and unilobate cirrophore (neuropodial superior not developed). Dorsal and ventral cirrostyles cirriform, smooth (without papillae), elongated (Fig 5F–H); dorsal cirrostyle extending beyond dorsal ligule, as long as two-thirds of dorsal cirrophore in anterior natatory chaetigers (Fig 5F), becoming markedly shorter in medial natatory chaetigers (up to one-third of dorsal cirrophore; Fig 5G, H), subequal in posterior chaetigers (Fig 5H); ventral cirrostyle extending markedly beyond ventral ligule, as long as two-fifths to one-third of dorsal cirrophore in anterior and medial natatory chaetigers (Fig 5F–G), as long as three-quarters in posterior natatory chaetigers (Fig 5H). Dorsal cirrophore expanded, sub-rectangular, distally with subequal, tongue-shaped upper and lower lamellae, more distinct in medial natatory chaetigers; two divergent vessels running lengthwise (Fig 5G). Dorsal ligule fusiform, elongated, emerging basally from one-quarter to one-third of

lower edge of dorsal cirrophore in all natatory chaetigers (Fig 5F–H); dorsal ligule extending markedly beyond median ligule in anterior and medial natatory chaetigers, subequal in posterior natatory chaetigers. Notopodial prechaetal lobe and median ligule non-modified, slender, tapering, nearly subequal but lobe shorter (Fig 5F–H). Neuroacicular ligule elongated, slender, markedly shorter than ventral ligule. Neuropodial postchaetal lobe with upper lamella from parapodia 32, broadly rhomboid in parapodia 32–35 (Fig 5F), markedly expanded, asymmetrical, cordiform with distal tip in medial natatory chaetigers (Fig 5G), shorter and leaf-like in posterior natatory chaetigers (Fig 5H). Inferior lobe slightly enlarged in medial natatory chaetigers. Ventral ligule non-modified, slender, tapering, longer than neuroacicular ligule in chaetigers in anterior and medial natatory chaetigers (Fig 5F, G), shorter in posterior natatory chaetigers (Fig 5H). Ventral cirrophore, markedly elongated, acuminate, with drop-shaped lower lobe only (elfin-shoe shaped; Fig 5F–H), more distinct in anterior and medial natatory chaetigers.

Post-natatory region lost in holotype, paratype (Fig 3E) with unmodified notopodia, consisting of dorsal cirri with cirrostyle and cirrophore, dorsal ligule, notopodial prechaetal lobe, and median ligule; neuropodia consisting of neuroacicular ligule with postchaetal lobes, ventral ligule, and ventral cirri with cirrostyle and cirrophore (neuropodial inferior and superior lobes not developed). Dorsal and ventral cirrostyles cirriform, smooth; dorsal cirrostyle extending slightly beyond dorsal ligule, as long as dorsal cirrophore, becoming posteriorly markedly longer (up to 2-3 times length of dorsal cirrophore); ventral cirrostyle short, extending to base of ventral ligule, becoming posteriorly longer. Dorsal and ventral cirrophores becoming reduced posteriorly. Parapodial ligules long, slender, tapering; dorsal ligule longer than median ligule. Notopodial prechaetal lobe short, slender, tapering. Neuropodial postchaetal lobe conical, longer than neuroacicular ligule. Neuroacicular ligule slightly longer than ventral ligule.

Pre-pygidial region lost in holotype, paratype (Figs 3F, 5I) with markedly reduced parapodial projections except short dorsal and ventral cirrostyles and ventral ligule.

Aciculae mostly dark red; present throughout body except notoaciculae absent in first two chaetigers (Fig 5A); notoaciculae with proximal half as wide as neuroaciculae. Aciculae of two types: needle-shaped (Fig 5A, B, E-H) and hooked (Figs 3F, 5I, 7B, C). Needle-shaped aciculae present almost throughout except most posteriorly, with basal end barely expanded in natatory chaetigers (Fig 7A). Hooked aciculae coarse, protruding distinctly from body surface, with distal end directed upwards (Fig 5I), present only in pre-pygidial chaetigers (Fig 7B, C); notoaciculae stouter and more curved (Fig 5B) than neuroaciculae (Fig 5C).

Figure 7. *Pectinereis strickrotti* gen. nov., sp. nov. male chaetae and female parapodia. A, D. Holotype (SIO-BIC A9836), epitokous male: A, barely expanded proximal end of notoaciculae; D, homogomph falcigers, neuropodial subacicular fascicle (chaetiger 15). B, C, E. Paratype (SIO-BIC A9889), epitokous male: B, strongly hooked notoacicula (most posterior chaetigers); C, hooked neuroacicula (most posterior chaetigers); E, ensiform neurochaetae from mid-natatory chaetigers. F, G. Paratype (SIO-BIC A9891), mature female: F, parapodium from possibly anterior region in anterior view; G, same parapodium in posterior view. Abbreviation: po, notopodial postchaetal lobe. Scale bars: A, B, 0.2 mm; C, 0.1 mm; D, E, 50 µm; F, G, 1 mm. Credits: A, D, Tulio F. Villalobos; B, C, E-G, Greg Rouse.

Notochaetae consisting of homogomph spinigers and ensiform chaetae (Fig 7E); homogomph spinigers present in all pre-natatory chaetigers. Upper and lower neurochaetae consisting of homogomph spinigers, homogomph falcigers, and ensiform chaetae; both spinigers and falcigers present in all pre-natatory chaetigers. Ensiform epitokous chaetae replacing all atoke chaetae in notopodia and neuropodia in all natatory chaetigers from chaetiger 32.

Blades of homogomph spinigers finely serrated, with teeth evenly spaced; long, with high b/a ratio. Blades of homogomph falcigers spiniger-like (Fig 7D); long (b/a ratio: 6.8–

7.5), with blunt falcate tip, lacking incurved terminal tooth, entirely serrated, with slender serrations, pointing upwards, with sub-distal serrations growing laterally to falcate tooth.

Pygidium lost in holotype, metamorphosed in complete paratype (Fig 3E, F). Pygidial rosette with 12 long, dorsal, digitiform, subdistal papillae (Fig 3F); anal cirri not present.

Etymology

The species is named in honor of Bruce Strickrott, Group Manager and lead submersible pilot of the DSV *Alvin* (Woods Hole Oceanographic Institution), who chased these worms for many years before finally succeeding in their skillful capture.

Remarks

Pectinereis strickrotti gen. nov., sp. nov. is described based on epitokous males from off Costa Rica at about 1,000 m depth (Fig 1A, B, D, E) and a small mid-anterior fragment of an infaunal mature female (Fig 1C) sequenced for molecular analysis. As well as the DNA sequence of *COI* (Fig 2A; GenBank OQ415955), the few parapodia of this female (Figs 1C, 7F, G) are comparable with the other individuals of *P. strickrotti* gen. nov., sp. nov. (Fig 5E). They consisted of well-developed and slender ligules (dorsal, median, ventral, and neuroacicular) and notopodial prechaetal lobe, blunt neuropodial postchaetal, inferior and notopodial postchaetal lobes, and short, cirriform dorsal and ventral cirri (Fig 5F, G). Although this fragment has well-developed ova (~350 µm in diameter), it is uncertain if the female undergoes epitokal metamorphosis because it appears to correspond to an anterior-middle region, where no traces of distinct modifications are detected in epitokous nereidids. The holotype and three paratypes differed by a maximum of five bases (Fig 2A) across the 576 *COI* bases sequenced for all specimens, an uncorrected distance of less than 1%. An epitoke was also seen (Fig 1E; S2 Video) near the type locality in January 2019 at the Parrita Scar methane seep (8.951° N; 84.634° W) at ~1,100 m on dive S0218 of the ROV *SuBastian*. The animal was collected via slurp but escaped before reaching the surface.

Type locality

Close to summit of Mound 12, near methane seeps at ~1,000 m depth (8.929° N; 84.313° W), off the Pacific coast of Costa Rica.

Biology

The swimming worms collected in this study were epitokous males. One of them spawned out all the sperm after removing tissue of most-posterior end for molecular purposes. One incomplete atokous female with well-developed ova (~350 µm diameter) was found dwelling in the sediment (Fig 1C). We can infer that only the males become epitokes and the females remain in the sediment, as occurs in species of *Alitta* such as *A. grandis* [6, 108, 129] and possibly *Websterinereis glauca* (Claparède 1870) [114].

Habitat

Near methane seeps; ranging ~1,000−1,100 m depth.

Distribution

Species known only from type locality.

Discussion

In the present study, we established a new nereidid genus and species, *Pectinereis strickrotti* gen. nov., sp. nov., from the deep sea off the Pacific coast of Costa Rica using both morphological features and genomic evidence. This is the first endemic deep-sea nereidid genus established in over 140 years. We described its morphology in detail, obtained newly-generated mitogenomes from this and two other species, and comparatively analyzed them with other members. We also determined its phylogenetic position within the family Nereididae.

Morphology

The epitokous specimens of *Pectinereis strickrotti* gen. nov., sp. nov. show unusual morphology among nereidids by the presence of four autapomorphic features: 1) pectinate branchiferous parapodial cirrostyles, 2) hooked aciculae, 3) elfin-shoe shaped ventral cirrophores, and 4) elongate, fusiform dorsal ligule emerging basally to expanded cirrophores. Each diagnostic characters makes the new genus unique within the family, as demonstrated in the phylogenetic analyses (Fig 2B, S1 Fig).

Nowadays, it is uncommon to use typical epitokal morphology to establish genera within Nerididae, possibly because it differs even among congeneric species. Several genera (e.g., *Nereilepas* de Blainville, 1828, *Heteronereis* Örsted, 1843, *Naumachius* Kinberg, 1865, *Nicomedes* Kinberg, 1865, *Iphinereis* Malmgren, 1865, *Hedyle* Malmgren, 1867) were earlier proposed based upon epitokal features, such as the hypertrophied prostomium, the distinct body regions, and the appearance of lamellae and cultrate chaetae [95–99], but were later synonymized as they represented reproductive forms of members of other already known genera [100–102]. Nonetheless, Chamberlin [101] considered sufficient the presence of two highly peculiar epitokal features to propose his new genus *Kainonereis* Chamberlin, 1919: elytriform dorsal cirri and single anastomosed antennae. Recently, Conde-Vela *et al.* [103] re-evaluated *Kainonereis* and confirmed the distinctive epitoke metamorphosis of those elytra-like dorsal cirri as valuable to distinguish among genera, while the basally fused antennae were regarded as a specific feature of *K. alata* Chamberlin, 1919.

The four distinctive features, either separately or altogether, of the remarkable deep-sea nereidid from Costa Rica and the distinct phylogenetic placement justify the new genus and species. The morphology of these features is discussed below, whereas the detailed comparison with other closely related genera was addressed in the new genus remarks.

1) Branchiae. Oxygen exchange in nereidids is taken over by the branchiae, which are present as vascularized parapodial structures [1]. Elaborate branchiae (gills) develop only in a

few genera; they are associated with the dorsal cirrophore and start some distance from the prostomium. The most distinctive and complex gills have been reported in two genera: *Dendronereides* Southern, 1921, and *Dendronereis* Peters, 1854. The gills in *Dendronereides* are arborescent with branched bunches of filaments inserted basally on the cirrophore and above the dorsal ligule. In contrast, those in *Dendronereis* Peters, 1854 are markedly modified dorsal cirrophores with bipinnate shapes, consisting of branches arising laterally from the primary axis. Enlarged dorsal cirrophores have also been reported as gills in *Gymnonereis* [104], *Namalycastis* [89, 105], *Nereis* [106], and *Tambalagamia* Pillai, 1961 [107], and those highly vascularized should also be treated in the same manner in members of *Alitta* (see [49]: Figs. 2F, 3E; [108]: Figs. 3E-G, 9F, 17K, 18G), *Nectoneanthes* ([109]: Figs. 8D, 11D, 14D), *Stenoninereis* ([90]: Figs. 2D, 5D), as well as some *Neanthes* with enlarged dorsal cirrophores (see [110]: table II, as shape of dorsal ligule), among others.

The gills in *Pectinereis* gen. nov. are an exceptional case among nereidids. These respiratory organs are modified dorsal and ventral cirrostyles of anterior chaetigers, with the former thicker and encompassing a slightly higher range of chaetigers. They are pectinate in shape by the 4–11 filaments that arise from the upper edge and vascularized with a main broad blood vessel that runs along the stem and branches out to the filaments. In contrast to nereidids with gills associated to dorsal cirrophores and starting at some distance from the prostomium, these structures in *Pectinereis* gen. nov. are associated not only with the dorsal but also to the ventral cirrostyles present from the first to some anterior parapodia.

Pectinereis gen. nov. gills may remind the shape of scalloped or swollen cirrostyles of some epitokous nereidids, which are associated with the chemical reception of pheromones [111, 112]. Nevertheless, the former are short, knob-like structures exclusive of natatory parapodia present typically in males, whereas those in *Pectinereis* gen. nov. are elongate and digitiform, restricted to the pre-natatory parapodia. Although the swollen cirrostyles of

epitokous nereidids and the pectinate cirrostyles of *Pectinereis* gen. nov. are present in both dorsal and ventral cirri of pre-natatory parapodia, the forms are very different by themselves. Additionally, the swollen cirrostyles are present in up to the first 7-8 parapodia, whereas the pectinate cirrostyles of *Pectinereis* gen. nov. are present at least in the first 14 parapodia. These structures are not homologs due to their morphology and function and should not be mistaken.

2) Aciculae. These powerful supportive chaetae in nereidids are deeply embedded within the parapodia so that only sometimes its small tip emerges from the body surface. The typical aciculae have a billiard cue shape — gradually tapering towards the distal end, straight, slender, with a truncate proximal end— sometimes slightly curved, particularly in epitokous parapodia; however, in mature *Tambalagamia fauveli* Pillai, 1961 they are sharply curved at the tip with a marked sickle shape in natatory chaetigers [6].

Remarkably, some of the aciculae in *Pectinereis* gen. nov. differ from all the forms previously recorded in nereidids. Although the aciculae present are mainly of the typical form, those shown in the notopodia and neuropodia of most posterior chaetigers (pre-pygidial region), located just immediately before the pygidial rosette —a sex-specific epitokal structure for releasing the sperm through developed papillae [113–115]— have a more robust and stouter appearance with a falcate distal end, exhibit a curved body that tapers towards a blunt proximal end, and protrude conspicuously beyond the parapodial surface. All these features of the ‘hooked aciculae’ are generally more pronounced in the notopodia. They seem to resemble the sickle-shaped aciculae of *T. fauveli* epitokes, although they differ in form and appearance on the body. For instance, the hooked aciculae in *Pectinereis* gen. nov. in the post-natatory parapodia are stouter and curved with a falcate distal end, whereas the sickle-shaped aciculae of *T. fauveli* are slender, straight, with a sharply curved tip, and restricted to the natatory parapodia.

Pectinereis gen. nov. hooked aciculae presumably develop during male sexual maturation before spawning. The appearance of specialized chaetae within epitokous nereidids is a frequent phenomenon. It generally involves the emergence of specific natatory compound chaetae to facilitate swimming within the water column for swarming, which depending on the species, can be paddle-like [114, 116], capillaries [117] or ensiform [108]. However, quite different chaetae have rarely been reported within nereidids at maturity. These are hook-shaped non-acicular chaetae ornamented with dorsal spines and forming part of the neuropodial bundle of the third chaetiger in some *Micronereis* species (see [118]: Figs. 9, 25, 30). These specialized chaetae have been suggested as male genital structures with supporting copulatory functions that are probably used to pierce the epidermis of the posterior dorsal surface of the female for an eventual sperm transfer [118–120]. Analogous chaetae have been found mainly in the meiofaunal syllid *Sphaerosyllis hermaphrodita* Westheide, 1990, where the aciculae of a single chaetiger are modified as a solid and straight copulatory structure with a curved and blunt distal end, subdistally broadened and flattened, and ornamented with teeth [121, 122].

The presence and form of genital chaetae in a few epitokous male species of Nereididae and Syllidae and the location and form of the posteriormost aciculae in *Pectinereis* gen. nov. may provide some hints on the tentative function of hooked aciculae present in the reproductive males of the new genus. However, their reliable reproductive purpose has yet to be discovered, and we can only provide conjectures based on the backgrounds mentioned above. We infer that the epitokous males of *Pectinereis* gen. nov. might produce mechanical body wall ruptures on the fully mature female through the hooked aciculae to discharge the sperm immediately after through the pygidial papillae. A detailed study of the reproductive strategies in this species, however, is needed to support this hypothesis.

3) Ventral cirrophores. Nereidids have ventral cirri consisting of two main components: (1) the distal, usually elongated cirrostyle, and (2) the proximal, frequently barely developed cirrophore. These ventral cirrophores are characteristic in atokous nereidids; however, epitoke worms are typically well-developed, highly vascularized, enlarged, and divided into upper and lower lamellae in natatory parapodia. The upper lamella is generally less developed and may present an additional secondary flap, unlike the single, reniform, foliose lower lamellae. In the pre-epitokous parapodia of *Pectinereis* gen. nov., the ventral cirrophores show the typical atokous form, although, in the natatory chaetigers, they are notably different from other epitokous nereidids. They are markedly elongated, acuminate, lacking an upper lobe but with a drop-shaped lower lobe, whose overall form is reminiscent of an elf's shoe. This elfin-shoe shaped ventral cirrophore has yet to be documented within Nereididae previously. It is, therefore, uncertain whether this morphological adaptation is related to the deep-sea environment.

4) Dorsal ligules. When present in nereidids, the dorsal ligule is attached frontally to the dorsal cirrophore, although, in *Stenoninereis* species, it seems to be attached to the notoacicular ligule itself instead of the dorsal cirrophore (see [90]). The division between the dorsal ligule and the cirrophore appears as an intermediate constriction that runs typically from the dorsal cirrostyle towards the base of the ligule (see [4]: Fig 1C).

When the cirrophore enlarges progressively, the dorsal ligules change size, location, or both. Therefore, the following combinations are usually recognizable in nereidids with dorsal cirrophores enlarging towards posterior end: (1) dorsal ligule of similar size throughout body but located distally in posterior parapodia, with base occupying more or less half of cirrophore, as occurs in some *Neanthes* (see [123]: Pl. 3, Figs. 12–14) and *Perinereis* (see [124]: Figs. 13, 16) species; (2) dorsal ligule becoming strongly reduced and located distally in posterior parapodia, with base occupying a small part of cirrophore, as occurs in

Ceratonereis (see [125]: 4M, 6H), *Pseudonereis* (see [126]: 9K; [127]: 2E, F) or *Alitta succinea* group species (see [49]: Fig 2E, F); and (3) dorsal ligule becoming enlarged, with base occupying all the frontal flank of cirrophore, as shown in *Cheilonereis* (see [128]: Fig 6F, G), *Nectoneanthes* (see [109]: Fig 11C, 13D), and *Alitta virens* group (see [108]: Fig 7G, 17K) species. However, an additional form can be recognized solely in *Pectinereis* gen. nov.: (4) dorsal ligule of similar size throughout the body but located sub-basally in posterior parapodia, with base occupying a small part of cirrophore, giving a false impression of the cirrophore being 'bifurcated.' This fourth combination of dorsal cirrophore-dorsal ligule also makes the new genus unique among nereidids.

Phylogeny and systematics

The phylogenetic reconstructions shown here were inferred from a three-gene analysis (S1 Fig) and the complete newly obtained mitogenomes of three species of nereidids plus other sequences retrieved from GenBank (Fig 2B). The latter analysis showed a general agreement with the previous mitogenomic assessment of Nereididae [67], supporting the current non-monophyletic status of Nereidinae de Blainville, 1818 and Gymnonereidinae Banse, 1977. The type species of *Paraleonnates* (*P. uschakovi* Chlebovisch & Wu, 1962), a supposed member of Nereidinae (see [6]), showed a variable position (Fig 2B, S1 Fig), as did *Laeonereis*, which was either sister group to Nereidinae or nested inside, when it is typically referred to as a member of Gymnonereidinae (see [130]). Despite both genera being the subject of recent comprehensive morphological studies (e.g., [121, 131]), none have dealt with their current subfamily position. Among the subfamilies, Nereidinae is presently the most difficult to diagnose since its membership is broader and much more heterogeneous and speciose than the others.

Gymnonereidinae was originally erected by Banse [132] for *Gymnonereis* Horst, 1919, *Ceratocephale* Malmgren, 1867, *Micronereides* Day, 1963 and *Tambalagamia* Pillai,

1961, due to the presence of two unique features among nereidids: (1) the anterior region carrying dense tufts of chaetae, and (2) the double neuropodial cirri. However, Fitzhugh [130] expanded it to include all genera without paragnaths, except Namanereidinae, encompassing thus a more complex group of taxa. Hylleberg & Nateewathana [133] and Khlebovich [6] followed Banse's original concept. Later, Santos et al. [134] restricted Gymnonereidinae *sensu* Banse [132] to the four original genera through a phylogenetic analysis based upon morphology mainly by the presence of double ventral cirri, dense chaetal bundles in anterior parapodia, and subacicular notopodial chaetae. On the contrary, both *Paraleonnates* and *Laeonereis* have single ventral cirri, and the chaetal bundles of the anterior parapodia do not differ significantly in density from the subsequent ones. This different morphology and the molecular tree based on mitochondrial genomes support that none of these two genera belong to Gymnonereidinae, in which *Laeonereis* was earlier proposed (see [130]) but is currently positioned as sistergroup to Nereidinae [135, this study].

Position of *Paraleonnates*

The genus consists presently of four species: *Paraleonnates bolus* (Hutchings & Reid, 1991), *P. sootai* (Misra, 1999), *P. tenuipalpa* (Pflugfelder, 1933), and the type species *P. uschakovi* Chlebovitsch & Wu, 1962. The genus has been typically considered within Nereidinae. Recently, morphology and molecular analysis suggested *Paraleonnates* does not belong in Nereidinae based on phylogenetic results and in having a Group II mitochondrial gene order, which differs from Group I by the position of three t-RNA genes encoding for tyrosine, methionine, and aspartic acid [61, 67, 134, 135]. In the present study, *P. uschakovi* was placed as the sister group to all other Nereidinae sampled here according to the three-gene tree (S1 Fig) or sister to the clade of Gymnonereidinae + Namanereidinae + Dendronereidinae based on the mitogenomic tree (Fig 2B). At present we concur with previous studies in not placing *Paraleonnates* within Nereidinae based on these conflicting

results. A critical morphological reassessment of *Paraleonnates* is needed as well as further molecular data from more taxa.

Paraleonnates species exhibit a unique feature among nereidids, namely that the maxillary ring of the proboscis has dorsally transverse rows of paragnaths and papillae. Contrary to the typical features present in the subfamily Nereidinae, *Paraleonnates* has a set of shared characters that makes its species distinctive: 1) prostomium anteriorly with a deep cleft, 2) glandular oesophageal caeca absent, 3) first two chaetigers with notoacicula, 4) three parapodial lobes present only in neuropodia (superior, inferior, and postchaetal), and 5) falcigers absent throughout. The first three features link *Paraleonnates* with *Ceratonereis* Kinberg, 1865 and *Solomononereis* Gibbs, 1971, two genera related to each other and presently treated as belonging to Nereidinae, although their phylogenetic position within the family is still uncertain — being part of Nereidinae according to morphology [134, 136], or doubtfully *Ceratonereis* as sister to all nereidids based on molecular markers [135]. However, *Ceratonereis* and *Solomononereis* lack a superior neuropodial lobe, present falcigers, and have different arrangement of paragnaths dorsally on the maxillary ring. On the other hand, the last three features can partially be shared with *Alitta*, *Nectoneanthes*, and a few *Neanthes* species with three neuropodial lobes. *Paraleonnates* shares with *Nectoneanthes* both the presence of notoacicula in the first two chaetigers —present in *Alitta* and absent in those *Neanthes* species— and the absence of falcigers throughout the body —absent in *Alitta* and *Neanthes* species. Nevertheless, *Paraleonnates* can be easily distinguished from the members of those three genera by the absence of a notopodial postchaetal lobe, the prostomium with an anterior cleft, and the absence of glandular caeca.

Accepting Dendronereidinae

In this study, we also confirmed Dendronereidinae Pillai, 1961 as valid. The subfamily was originally proposed to include the branchiae-bearing genera *Dendronereis* Peters, 1854,

Dendronereides Southern, 1921, and *Tambalagamia* Pillai, 1961 with the former as type [107]. However, as suggested previously [1, 91, 132, 134] and discussed above, the complex gills in members of those genera are not homologous. They are markedly modified, branched, bipinnate dorsal cirrophores in *Dendronereis*, arborescent tufts inserted basally between the cirrophore and the dorsal ligule in *Dendronereides*, and foliaceous dorsal cirrophore in *Tambalagamia*. The latter belongs to Gymnonereidinae due to its close morphological [132, 134] and molecular [67, 135] similarity to *Gymnonereis* and *Ceratocephale*. Also, *Dendronereis* is distinguishable from *Dendronereides* by having a ventral ligule (absent in *Dendronereides*) and in lacking notopodial glandular organs and compound falcigers (both present in *Dendronereides*). However, molecular studies of *Dendronereides* have yet to be undertaken.

Members of *Dendronereis* are unique among nereidids by the presence and type of branchiae restricted to some anterior chaetigers. Recently, a complete mitogenome was published for *Dendronereis chipolini* Hsueh, 2019, whose identity was confirmed by a nereidid specialist [66]. In the present results (Fig 2, Fig S1), the placement of *D. chipolini* was as sistergroup to an apparently misidentified specimen of the species published as *Neanthes glandicincta* [64], and, based on the sequence similarities, is likely also *D. chipolini*. The placement of *D. chipolini* supports the recognition of Dendronereidinae in contrast to its synonymy with Gymnonereidinae as previously proposed [130].

Although *Dendronereides* was not included in the present mitogenomic analysis, we suggest exclusion from Dendronereidinae because of its marked differences with *Dendronereis* and leaving it unplaced, resulting in the subfamily being monotypic as earlier proposed by Santos et al. [134]. However, its validity still needs to be addressed. *Dendronereides* is more like *Tylorrhynchus* —considered as belonging to Gymnonereidinae [130] but recently unplaced (see S1 Fig and [135])— rather than other nereidid genera mainly

because of the presence of papillae on both rings of the proboscis, dorsal cirrophores consisting of glandular organs with openings, compound falcigers, and the absence of ventral ligule. These genera require further morphological and molecular data to assess their placement.

Following the recent proposal of revisited definitions for the subfamilies Gymnonereidinae and Nereidinae [135], an emendation of Dendronereidinae is provided below considering the earlier and current knowledge on the morphology of the genus' members (e.g., [1, 91, 137−140]:

Dendronereidinae Pillai, 1961

Type genus. *Dendronereis* Peters, 1854.

Diagnosis (emended from Pillai [107])

Prostomium with anterior cleft. Two antennae. Palps with elongated palpophores and conical palpostyles. Tentacular belt length equal to or longer than length of chaetiger 1. Tentacular cirri with four pairs, distinct cirrophore present. Proboscis without paragnaths, papillae present on both rings; maxillary ring sometimes smooth. Paired esophageal caeca absent. Parapodia biramous, except first two chaetigers, uniramous (lacking notoacicula). Dorsal cirrophore of some anterior chaetigers divided into numerous branchial filaments. Parapodia of branchial chaetigers multilobed, including notopodial postchaetal, and superior, inferior, and postchaetal lobes. Dorsal, median, and ventral ligules present. Single dorsal and ventral cirri. Notochaetae and neurochaetae with homogomph spinigers only. Mitochondrial gene order of Group II type as identified by Park et al. [61].

Composition

One genus and five species: *Dendronereis aestuarina* Southern, 1921, *D. arborifera* Peters, 1854, *D. chipolini* Hsueh, 2019, *D. dayi* Misra, 1999, and *D. pinnaticirris* Grube, 1878.

Acknowledgements

We are grateful to the cruise party of AT42-03 for their help in collecting the specimens and to Marina McCowin and Charlotte Seid for initial sequencing and cataloging, respectively. The US National Science Foundation (NSF-OCE 0939557) supported this research. We also appreciate the generosity of Masaroni Sato (University of Kagoshima) in sharing fresh *Nectoneanthes* specimens for sequencing. TFVG was supported by CONACYT under the Postdoctoral Stays for Researchers in Mexico grant and appreciated the kind support of Omar Valencia Méndez (CICESE) by providing facilities and installations during the writing process of this contribution.

References

1. Bakken T, Glasby CJ, Santos CSG, Wilson RS. Nereididae Blainville, 1818. In: Westheide W, Purschke G, Böggemann M, editors. Handbook of zoology. A natural history of the Phyla of the Animal Kingdom, Annelida: Polychaetes. Ösnabruck: De Gruyter; 2022. pp. 259–307.
2. Read G, Fauchald K. Nereididae Blainville, 1818. World Polychaeta Database. 2023 Mar 12. Available from: https://www.marinespecies.org/polychaeta/aphia.php?p=taxdetails&id=22496.
3. de León-González JA, Villalobos-Guerrero TF, Conde-Vela VM. Nereididae de Blainville, 1818. In: de León-González JA, Bastida-Zavala JR, Carrera-Parra LF, García-Garza ME, Salazar-Vallejo SI, Solís-Weiss V, et al., editors. Marine Annelids

from Mexico and Tropical America. Monterrey: Universidad Autónoma de Nuevo León; 2021. pp. 453–488. [In Spanish].

4. Villalobos-Guerrero TF, Conde-Vela VM, Sato M Review of *Composetia* Hartmann-Schröder, 1985 (Annelida: Nereididae), with the establishment of two new similar genera. J Nat Hist. 2021;55(37–38): 2313–2397. doi: 10.1080/00222933.2021.1976295
5. Wu BL, Sun R, Yang DJ. The Nereidae (polychaetous annelids) of the Chinese coast. Beijing: China Ocean Press; 1985.
6. Khlebovich VV. Fauna of Russia and neighboring countries. Polychaetous Annelids, Volume III. Polychaetes of the family Nereididae of the Russian seas and the adjacent waters. Proc Zool Inst Russ Acad Sci. 1996;140: 1–221. [in Russian]
7. Sato M. Nereididae (Annelida) in Japan, with special reference to life-history differentiation among estuarine species. In: Motokawa M, Kajihara H, editors. Species Diversity of Animals in Japan. Tokyo: Springer Japan; 2017. pp. 477–512. doi: 10.1007/978-4-431-56432-4_19.
8. Glasby CJ, Erséus C, Martin P. Annelids in extreme aquatic environments: diversity, adaptations and evolution. Diversity. 2021;13(2), 98. doi: 10.3390/d13020098.
9. Glover AG, Higgs N, Horton T. World Register of Deep-Sea species (WoRDSS). 2022 Sep 03. Available from https://www.marinespecies.org/deepsea. doi: 10.14284/352.
10. Blake JA. Polychaeta from the vicinity of deep-sea geothermal vents in the eastern Pacific. I: Euphrosinidae, Phyllodocidae, Hesionidae, Nereididae, Glyceridae, Dorvilleidae, Orbiniidae and Maldanidae. Bull Biol Soc Wash. 1985; 6: 67–101.
11. Fauchald K. Benthic polychaetous annelids from deep water off western Mexico and adjacent areas in the Eastern Pacific Ocean. Allan Hancock Monogr Mar Biol. 1972;7: 1–575.

12. Fauchald K. Some nereid polychaetes from deep water. In: Reish DJ, Fauchald K, editors. Essays on polychaetous annelids in memory of Dr. Olga Hartman. Los Angeles: The Allan Hancock Foundation, University of Southern California; 1977. pp. 157–171.
13. Levin LA, Orphan VJ, Rouse GW, Rathburn AE, Ussler W, Cook GS, et al. A hydrothermal seep on the Costa Rica margin: middle ground in a continuum of reducing ecosystems. Proc R Soc B. 2012;279: 2580–2588. doi: 10.1098/rspb.2012.0205.
14. Shimabukuro M, Santos CSG, Alfaro-Lucas JM, Fujiwara Y, Sumida PY. A new eyeless species of *Neanthes* (Annelida: Nereididae) associated with a whale-fall community from the deep Southwest Atlantic Ocean. Deep Sea Res 2 Top Stud Oceanogr. 2017;146: 27–34. doi: 10.1016/j.dsr2.2017.10.013.
15. Drennan R, Wiklund H, Rabone M, Georgieva MN, Dahlgren TG, Glover AG. *Neanthes goodayi* sp. nov. (Annelida, Nereididae), a remarkable new annelid species living inside deep-sea polymetallic nodules. Eur J Taxon. 2021;760: 160–185. doi: 10.5852/ejt.2021.760.1447.
16. Lejzerowicz F, Gooday AJ, Barrenechea Angeles I, Cordier T, Morard R, Apothéloz-Perret-Gentil L, et al. Eukaryotic biodiversity and spatial patterns in the Clarion-Clipperton zone and other abyssal regions: insights from sediment DNA and RNA metabarcoding. Front Mar Sci 2021;8: 671033. doi: 10.3389/fmars.2021.671033.
17. Glover AG, Smith CR, Paterson GLJ, Wilson GDF, Hawkins L, Sheader M. Polychaete species diversity in the central Pacific abyss: local and regional patterns, and relationships with productivity. Mar Ecol Prog Ser. 2002;240: 157–170. doi: 10.3354/meps240157.
18. Alalykina I. Polychaeta: A review on the deep-sea benthic polychaetes along the NW Pacific. In: Saeedi H, Brandt A, editors. Biogeographic Atlas of the Deep NW Pacific Fauna. Advanced Books; 2020. pp: 175–212.

19. Gunton LM, Kupriyanova EK, Alvestad T, Avery L, Blake JA, Biriukova O, et al. Annelids of the eastern Australian abyss collected by the 2017 RV 'Investigator' voyage. ZooKeys. 2021;1020: 1–198. doi: 10.3897/zookeys.1020.57921.
20. Grassle JF, Maciolek NJ. Deep-sea species richness: regional and local diversity estimates from quantitative samples. American Naturalist. 1992;139: 313–341. doi: 10.1086/285329.
21. Langeneck J, Busoni G, Aliani S, Lardicci C, Castelli A. Distribution and diversity of polychaetes along a bathyal escarpment in the western Mediterranean Sea. Deep Sea Res A. 2019;144: 85–94. doi: 10.1016/j.dsr.2019.01.006.
22. Hutchings P, Lavesque N. I know who you are, but do others know? Why correct scientific names are so important for the biological sciences. Zoosymposia. 2020;19: 151–163. doi: 10.11646/zoosymposia.19.1.16.
23. Hartman O. Systematic account of some marine invertebrate animals from the deep basins off southern California. Allan Hancock Pac Exped 1960;22(2): 69–216, plates 1–19.
24. Hartman O. Deep-water benthic polychaetous annelids off New England to Bermuda and other North Atlantic areas. Occ Pap Allan Hancock Fdn. 1965;28: 1–384.
25. Hartman O. Polychaetous annelids collected by the USNS Eltanin and Staten Island cruises, chiefly from Antarctic Seas. Allan Hancock Monogr Mar Biol. 1967;2: 1–387.
26. Hartmann-Schröder G. Die Polychaeten des Eulitorals. In: Hartmann-Schröder G, Hartmann G. Zur Kenntnis des Eulitorals der chilenischen Pazifikküste und der argentinischen Küste Südpatagoniens unter besonderer Berücksichtigung der Polychaeten und Ostracoden. Mitt Hamb Zool Mus Inst. 1962;60: 57–270.

27. Hartmann-Schröder G. Zur Kenntnis des Sublitorals der chilenischen Küste unter besonderer Berücksichtigung der Polychaeten und Ostracoden. II Die Polychaeten des Sublitorals. Mitt Hamb Zool Mus Inst. 1965;62(suppl): 59–305.
28. Hartmann-Schröder G. Polychaeten der Iberischen Tiefsee, gesammelt auf der 3. Reise der Meteor im Jahre 1966. Mitt Hamb Zool Mus Inst. 1975; 72: 47–73.
29. Hartmann-Schröder G. Zur Kenntnis des Eulitorals der australischen Küsten unter besonderer Berücksichtigung der Polychaeten und Ostracoden. Teil 6. Die Polychaeten der tropischsubtropischen Westküste Australiens (zwischen Exmouth im Norden und Cervantes im Süden). Mitt Hamb Zool Mus Inst. 1981;78: 19–96.
30. Day JH. The Polychaete fauna of South Africa. Part 8: New species and records from grab samples and dredgings. Bull Br Mus Zool. 1963;10(7): 383–445.
31. Hartman O, Fauchald K. Deep-water benthic polychaetous annelids off New England to Bermuda and other North Atlantic Areas. Part II. Allan Hancock Monogr Mar Biol. 1971;6: 1–327.
32. Blake JA, Hilbig B. Polychaeta from the vicinity of deep-sea hydrothermal vents in the Eastern Pacific Ocean. II. New species and records from the Juan de Fuca and Explorer Ridge systems. Pacific Science. 1990;44(3): 219–253.
33. Kirkegaard JB. Bathyal and abyssal polychaetes (errant species). Galathea Report. 1995;17: 7–56.
34. de León-González JA, Salazar-Vallejo SI. Four new nereidid species (Annelida, Polychaeta) collected during the MUSORSTOM cruises in the Indo-Pacific Ocean. Zoosystema. 2003;25(3): 365–375.
35. Imajima M. Deep-sea benthic polychaetes off Pacific coast of the northern Honshu, Japan. In: Fujita T, editor. Deep-sea fauna and pollutants off Pacific Coast of northern Japan. Nat Sci Mus Monogr. 2009;39: 39–192.

36. Wang Y, Cheng H, Wang C. A new eyeless species of *Nicon* (Annelida: Nereididae) from the deep Northwest Pacific Ocean. Acta Oceanol Sin. 2021;40(12): 20–26. doi: 10.1007/s13131-021-1886-z.
37. Glasby CJ, Fiege D, Van Damme K. Stygobiont polychaetes: notes on the morphology and the origins of groundwater *Namanereis* (Annelida: Nereididae: Namanereidinae), with a description of two new species. Zool J Linn Soc. 2014;171: 22–37. doi: 10.1111/zoj.12130.
38. Conde-Vela VM. The troglomorphic adaptations of Namanereidinae (Annelida, Nereididae) revisited, including a redescription of *Namanereis cavernicola* (Solís-Weiss & Espinasa, 1991), and a new Caribbean species of *Namanereis* Chamberlin, 1919. Subterranean Biology. 2017;23: 19–46. doi: 10.3897/subtbiol.23.13701.
39. Alves PR, Glasby CJ, Santos CSG. On the use of troglomorphic characters in Namanereidinae (Annelida; Nereididae) systematics. Zootaxa. 2018;4531(2): 195–210. doi: 10.11646/zootaxa.4531.2.2.
40. Danielopol DL, Baltanás A, Bonaduce G. The darkness syndrome in subsurface-shallow and deep-sea dwelling Ostracoda (Crustacea). Biosyst Ecol Ser. 1996;11: 123–143.
41. Martínez A, Di Domenico M, Worsaae K. Gain of palps within a lineage of ancestrally burrowing annelids (Scalibregmatidae). Acta Zoologica. 2014;95: 421–429. doi: 10.1111/azo.12039.
42. Iliffe TM, Wilkens H, Parzefall J, Williams D. Marine lava cave fauna: composition, biogeography, and origins. Science. 1984;225: 309–311.
43. Gonzalez BC, Martínez A, Borda E, Iliffe TM, Eibye-Jacobsen D, Worsaae K. Phylogeny and systematics of Aphroditiformia. 2018;34: 225–259. doi: 10.1111/cla.12202.

44. Gonzalez BC, Worsaae K, Fontaneto D, Martínez A. Anophthalmia and elongation of body appendages in cave scale worms (Annelida: Aphroditiformia). Zool Scr. 2018;47: 106–121. doi: 10.1111/zsc.12258.

45. Gonzalez BC, Martínez A, Worsaae K, Osborn K. Morphological convergence and adaptation in cave and pelagic scale worms (Polynoidae, Annelida). Sci Rep. 2021;11: 10718. doi: 10.1038/s41598-021-89459-y.

46. Sato M, Nakashima A. A review of Asian *Hediste* species complex (Nereididae, Polychaeta) with descriptions of two new species and a redescription of *Hediste japonica* (Izuka,1908). Zool J Linn Soc. 2003;137(3): 403–445. doi: 10.1046/j.1096-3642.2003.00059.x.

47. Glasby CJ, Wei NWV, Gibb KS. Cryptic species of Nereididae (Annelida: Polychaeta) on Australian coral reefs. Invertebr Syst. 2013;27(3): 245–264.

48. Sampértegui S, Rozbaczylo N, Canales-Aguirre CB, Carrasco F, Hernández CE, Rodríguez-Serrano E. Morphological and molecular characterization of *Perinereis gualpensis* (Polychaeta: Nereididae) and its phylogenetic relationships with other species of the genus of the Chilean coast, Southeast Pacifc. Cah Biol Mar. 2013;54: 27–40.

49. Villalobos-Guerrero TF, Carrera-Parra LF. Redescription of *Alitta succinea* (Leuckart, 1847) and reinstatement of *A. acutifolia* (Ehlers, 1901) n. comb. based upon morphological and molecular data (Polychaeta: Nereididae). Zootaxa. 2015;3919: 157–178. doi: 10.11646/zootaxa.3919.1.7

50. Park T, Kim W. A taxonomic study on *Perinereis nuntia* species group (Polychaeta: Nereididae) of Korea. Korean J Syst Zool. 2007;23: 75–85.

51. Park T, Kim W. Description of a new species for Asian populations of the cosmopolitan *Perinereis cultrifera* (Annelida: Nereididae). Zool Sci. 2017;34(3): 252–260. doi: 10.2108/zs160154

52. Wäge J, Valvassori G, Hardege JD, Schulze A, Gambi MA. The sibling polychaetes *Platynereis dumerilii* and *Platynereis massiliensis* in the Mediterranean Sea: are phylogeographic patterns related to exposure to ocean acidification?. Mar Biol. 2017;164: 199. doi: 10.1007/s00227-017-3222-x.
53. Kara J, Macdonald AHH, Simon CA. Integrative taxonomic methods reveal an incorrect synonymisation of the South African *Pseudonereis podocirra* (Schmarda) as the widespread *Pseudonereis variegata* (Grube) from Chile. Invertebr Syst. 2018;32: 1282–1297. doi: 10.1071/IS18016.
54. Kara J, Santos CSG, Macdonald AHH, Simon CA. Resolving the taxonomic identities and genetic structure of two cryptic *Platynereis* Kinberg species from South Africa. Invertebr Syst. 2020;34: 618–636. doi: 10.1071/IS19072.
55. Park T. DNA Barcoding of benthic ragworms of the genus *Nectoneanthes* (Polychaeta: Nereididae) collected in Korean waters. Anim Syst Evol Divers. 2021;37(3): 235–241. doi: 10.5635/ASED.2021.37.3.020.
56. Villalobos-Guerrero TF, Park T, Idris I. Review of some *Perinereis* Kinberg, 1865 (Annelida: Nereididae) species of Group 2 *sensu* Hutchings, Reid & Wilson, 1991 from the Eastern and South-eastern Asian seas. J Mar Biolog Assoc. 2021;101(2): 279–307. doi: 10.1017/S0025315421000126.
57. Villalobos-Guerrero TF, Kara J, Simon C, Idris I. Systematic review of *Neanthes* Kinberg, 1865 (Annelida: Errantia: Nereididae) from southern Africa, including a preliminary molecular phylogeny of the genus. Mar Biodivers. 2022;52: 21. doi: 10.1007/s12526-021-01244-2.
58. Liu M, Liu H, Wang Q, Guan S, Ge S. Phylogenetic relationships of twenty-one nereids species inferring two different evolutionary origins? Aquat Sci Technol. 2013;1(1): 167–180. doi:10.5296/ast.v1i1.2980.

59. Kim H, Jung G, Lee YC, Pae SJ, Kim CG, Lee YH. The complete mitochondrial genome of the marine polychaete: *Perinereis aibuhitensis* (Phyllodocida, Nereididae). Mitochondrial DNA A DNA Mapp Seq Anal. 2014;26(6): 869–870. doi: 10.3109/19401736.2013.861435.

60. Chen X, Li M, Liu H, Li B, Guo L, Meng Z, et al. Mitochondrial genome of the polychaete *Tylorrhynchus heterochaetus* (sic) (Phyllodocida, Nereididae). Mitochondrial DNA A DNA Mapp Seq Anal. 2015;27(5): 3372–3373. doi: 10.3109/19401736.2015.1018226.

61. Park T, Lee SH, Kim W. Complete mitochondrial genome sequence of the giant mud worm *Paraleonnates uschakovi* Khlebovich & Wu, 1962 (Polychaeta: Nereididae). Mitochondrial DNA Part B Resour. 2016;1(1): 640–642. doi: 10.1080/23802359.2016.1214552

62. Park HS, Nam SE, Rhee JS. Complete mitochondrial genome of the marine polychaete *Hediste japonica* (Phyllodocida, Nereididae). Mitochondrial DNA Part B Resour. 2020;5: 852–853.

63. Lin GM, Shen KN, Hsiao CD. Next generation sequencing yields the complete mitogenome of nereid worm, *Namalycastis abiuma* (Annelida: Nereididae). Mitochondrial DNA Part B Resour. 2016;1(1): 103–104. doi: 10.1080/23802359.2015.1137845.

64. Lin GM, Audira G, Hsiao CD. The complete mitogenome of nereid worm, *Neanthes glandicincta* (Annelida: Nereididae). Mitochondrial DNA Part B Resour. 2017;2(2): 471–472. doi: 10.1080/23802359.2017.1361346.

65. Nam SE, Lee S, Park TYS, Rhee JS. Complete mitochondrial genome of the marine polychaete, *Nereis zonata* (Phyllodocida, Nereididae) isolated from the Beaufort Sea.

Mitochondrial DNA Part B Resour. 2021;6(1): 231–233. doi: 10.1080/23802359.2020.1861994.

66. Zhen W, Wu X, Hao E, Xu W, Deng J, Zhu J, et al. The first complete mitochondrial genome of the genus *Dendronereis,* represented by *D. chipolini* Hsueh, 2019 (Annelida, Nereididae) from Beibu Gulf, China. Mitochondrial DNA B Resour. 2022;7(2): 393–395. doi: 10.1080/23802359.2022.2040389.
67. Alves PR, Halanych KM, Santos CSG. The phylogeny of Nereididae (Annelida) based on mitochondrial genomes. Zool Scr. 2020;49(3): 366–378.
68. Levin LA, Mendoza GF, Grupe BM, Gonzalez JP, Jellison B, Rouse GW, et al. Biodiversity on the rocks: macrofauna inhabiting authigenic carbonate at Costa Rica methane seeps. PLoS One. 2015;10(8): e0136129. doi: 10.1371/journal.pone.0131080.
69. Folmer O, Black M, Hoeh WR, Lutz RA, Vrijenhoek RC. DNA primers for amplification of mitochondrial cytochrome c oxidase subunit I from diverse metazoan invertebrates. Mol Mar Biol Biotechnol. 1994;3: 294–299.
70. Bolger AM, Lohse M, Usadel B. Trimmomatic: a flexible trimmer for Illumina sequence data. Bioinformatics. 2014;30(15): 2114–20.
71. Allio R, Schomaker-Bastos A, Romiguier J, Prosdocimi F, Nabholz B, Delsuc F. MitoFinder: Efficient automated large-scale extraction of mitogenomic data in target enrichment phylogenomics. Mol Ecol Resour. 2020;20(4): 892–905. doi: 10.1111/1755-0998.13160.
72. Li D, Luo R, Liu CM, Leung CM, Ting HF, Sadakane K, et al. MEGAHIT v1.0: A fast and scalable metagenome assembler driven by advanced methodologies and community practices. Methods [Internet]. 2016; 102:3–11. Available from: doi: 10.1016/j.ymeth.2016.02.020.

73. Laslett D, Canbäck B. ARWEN: a program to detect tRNA genes in metazoan mitochondrial nucleotide sequences. Bioinformatics. 2008;24(2): 172–175. doi: 10.1093/bioinformatics/btm573.

74. Bernt M, Donath A, Jühling F, Externbrink F, Florentz C, Fritzsch G, et al. MITOS: Improved de novo metazoan mitochondrial genome annotation. Mol Phylogenet Evol. 2013;69(2): 313–319. doi: 10.1016/j.ympev.2012.08.023.

75. Kearse M, Moir R, Wilson A, Stones-Havas S, Cheung M, Sturrock S, et al. Geneious Basic: An integrated and extendable desktop software platform for the organization and analysis of sequence data. Bioinformatics. 2012;28(12): 1647–1649. doi: 10.1093/bioinformatics/bts199.

76. Bushnell, B. BBMap. 2022; https://sourceforge.net/projects/bbmap/

77. Li H. Minimap2: pairwise alignment for nucleotide sequences. Bioinformatics. 2018;34(18):3094–3100. doi: 10.1093/bioinformatics/bty191

78. Li H. New strategies to improve minimap2 alignment accuracy. Bioinformatics. 2020;37(23):4572–4574. doi: 10.1093/bioinformatics/btab705

79. Danecek P, Bonfield JK, Liddle J, Marshall J, Ohan V, Pollard MO, et al. Twelve years of SAMtools and BCFtools. GigaScience. 2021;10(2): giab008. doi: 10.1093/gigascience/giab008

80. Leigh JW, Bryant D. POPART: full-feature software for haplotype network construction. Methods Ecol Evol. 2015;6(9):1110-6. doi: 10.1111/2041-210x.12410.

81. Templeton AR, Crandall KA, Sing CF. A cladistic analysis of phenotypic associations with haplotypes inferred from restriction endonuclease mapping and DNA sequence data. III. Cladogram estimation. Genetics. 1992;132:619–33.

82. Clement M, Posada D, Crandall KA. TCS: a computer program to estimate gene genealogies. Mol Ecol. 2000;9:1657–9.

83. Darriba Di, Posada D, Kozlov AM, Stamatakis A, Morel B, Flouri T. ModelTest-NG: A New and scalable tool for the selection of DNA and protein evolutionary models. Mol Biol Evol. 2020;37(1): 291–294. doi: 10.1093/molbev/msz189.
84. Edler D, Klein J, Antonelli A, Silvestro D. raxmlGUI 2.0: A graphical interface and toolkit for phylogenetic analyses using RAxML. Methods Ecol Evol. 2021;12: 373–377. doi: 10.1111/2041-210X.13512.
85. Maddison WP, Maddison DR. Mesquite: A modular system for evolutionary analysis. Version 3.61. 2019. Available from: http://mesquiteproject.org
86. Edgar RC. MUSCLE: Multiple sequence alignment with high accuracy and high throughput. Nucleic Acids Res. 2004;32: 1792–1797. doi: 10.1093/nar/gkh340
87. Villalobos-Guerrero TF. Redescription of two overlooked species of the *Perinereis nuntia* complex and morphological delimitation of *P. nuntia* (Savigny in Lamarck, 1818) from the Red Sea (Annelida, Nereididae). Zoosystema. 2019;41: 465–496. doi: 10.5252/zoosystema2019v41a24.
88. Nicoll PA. The anatomy and behavior of the vascular systems in *Nereis virens* and *Nereis limbata*. Biol Bull. 1954;106: 69–82.
89. Rasmussen E. *Namalycastis abiuma* (Müller in Grube, 1871), an aberrant nereidid polychaete in a Georgia salt marsh area with a survey of the faunal associations. Gulf Res Rep. 1994;9: 17–28.
90. Conde-Vela VM. Sinkhole and brackish water nereidid polychaetes: Revision of *Stenoninereis* Wesenberg-Lund, 1958 (Annelida). Subterr Biol. 2019;30: 95–115. doi: 10.3897/subtbiol.30.36273
91. Southern R. Polychaeta of the Chilka Lake and also of fresh and brackish waters in other parts of India. Indian Mus Calcutta Mem. 1921;5: 563–659.

92. Pettibone MH. Polychaeta Errantia of the Siboga Expedition. Part IV. Some additional polychaetes of the Polynoidae, Hesionidae, Nereidae, Goniadidae, Eunicidae, and Onuphidae, selected as new species by the late Dr. Hermann Augener with remarks on other related species. In: Weber M, Beaufort LF, Stock JH, editors. Siboga-Expeditie Uitkomsten op Zoologisch, Botanisch, Oceanographisch en Geologisch gebied verzameld in Nederlandsch Oost-Indië 1899-1900. Leiden: E.J. Brill; 1970. pp. 199–270.

93. Harper DE Jr. *Nereis* (*Neanthes*) *micromma* n. sp. (Polychaeta: Nereididae) from the northern Gulf of Mexico with a note on the structure of nereidid palps. Contr Mar Sci. 1979;22: 91–103.

94. Kaufmant C. Etude de l’appareil circulatoire de Nereis diversicolor (O.F. Müller) (Annelide Polychete). Diplome d’Etudes Superieures (Sciences Naturelles), Université de Lille. 1965. Available from: https://pepite-depot.univ-lille.fr/LIBRE/Th_Num/1965/50376-1965-87.pdf

95. de Blainville H. Vers, Dictionnaire des Sciences Naturelles. Strasbourg & Paris: F.G. Levrault; Volume 57, 1828.

96. Örsted AS. Grönlands Annulata dorsibranchiata. Kongel Danske Vidensk Selsk Skr, Naturvidensk Math Afd. 1843;10: 153–216.

97. Kinberg JGH. Annulata nova. Nereidum dispositio nova. Öfvers K VetenskAkad Förh Stockh. 1865;22: 167–179.

98. Malmgren AJ. Nordiska Hafs-Annulater. Öfvers K VetenskAkad Förh Stockh. 1865;22: 51–110.

99. Malmgren AJ. Annulata Polychaeta Spetsbergiae, Grönlandiae, Islandiae et Scandinaviae haetenus cognita. Helsingfors: Fronekell; 1867.

100. Ehlers E. Die Gattung *Heteronereis* (Oersted) und ihre Verhaeltnisse zu den Gattungen *Nereis* Gr. und *Nereilepas* Gr. Nachrichte der Koeniglichen Gesellschaft der Wisse schaften, Goettingen Math Phys Klasse. 1867;1867: 209–217.

101. Chamberlin RV. The Annelida Polychaeta [Albatross Expeditions]. Mem Mus Comp Zool Harv. 1919;48: 1–514.

102. Hartman O. The marine annelids erected by Kinberg with notes on some other types in the Swedish State Museum. Ark Zool. 1949;42A(1): 1–137.

103. Conde-Vela VM, Wu X, Salazar-Vallejo SI. Reevaluation and new species of *Kainonereis* Chamberlin, 1919 (Annelida: Polychaeta: Nereididae). Zool Stud. 2018;57: e6. doi: 10.6620/ZS.2018.57-06.

104. Hilbig B. Family Nereididae Johnston, 1845. In: Blake JA, Hilbig B, editors. Taxonomic atlas of the benthic fauna of the Santa Maria Basin and Western Santa Barbara Channel. 4 – The Annelida Part 1. Oligochaeta and Polychaeta: Phyllodocida (Phyllodocidae to Paralacydoniidae). California: Santa Barbara Museum of Natural History; 1994. pp. 301–327.

105. Feuerborn HJ. Eine Rhizocephale un zwei Polychaeten aus dem Süsswasser von Java und Sumatra. Verh Int Ver Theor Angew Limnol. 1931;5: 618–660.

106. Horst R. Three new *Nereis*-species from the Dutch East Indies. Zool Meded Leiden. 1919;5: 59–64.

107. Pillai TG. Annelida Polychaeta of Tambalagam Lake, Ceylon. Ceylon J Sci. 1961;4: 1–64.

108. Villalobos-Guerrero TF, Bakken T. Revision of the *Alitta virens* species complex (Annelida: Nereididae) from the North Pacific Ocean. Zootaxa. 2018;4483: 201–257. doi: 10.11646/zootaxa.4483.2.1.

109. Sato M. Resurrection of the genus *Nectoneanthes* Imajima, 1972 (Nereididae: Polychaeta), with redescription of *Nectoneanthes oxypoda* (Marenzeller, 1879) and description of a new species, comparing them to *Neanthes succinea* (Leuckart, 1847). J Nat Hist. 2013;47: 1–50. doi: 10.1080/00222933.2012.743609.

110. Villalobos-Guerrero TF, Idris I. Reproductive morphology and redescriptions of some *Neanthes* Kinberg, 1865 (Annelida: Nereididae) species from the southeastern Asian seas, with comparative synoptic tables of accepted species. Eur Zool J. 2021;88(1): 556–594. doi: 10.1080/24750263.2021.1899318.

111. Boilly-Marer Y. Etude expérimentale du comportement nuptial de *Platynereis dumerilii* (Annelida: Polychaeta): chémoréception, émission des produits génitaux. Mar Biol. 1974;24: 167–179. doi: 10.1007/BF00389352.

112. Boilly-Marer Y, Lassalle B. Electrophysiological responses of *Heteronereis* stimulated with sex pheromones (Annelida Polychaeta). J Exp Zool. 1978;205(1): 119–124. doi: 10.1002/jez.1402050114.

113. Herpin R. Comparaison entre le comportement sexuel de quelques Nereidiens des cotes de la Manche. C R Acad Sci, Paris. 1923;176: 536–539.

114. Clark RB. The origin and formation of the *Heteronereis*. Biol Rev Cambridge. 1961;36: 199–236. doi: 10.1111/j.1469-185X.1961.tb01584.x.

115. Bass NR, Brafield AR. The life cycle of the polychaete *Nereis virens*. J Mar Biol Ass UK. 1972;52: 701–726. doi: 10.1017/S0025315400021664.

116. Wilson RS. Family Nereididae. In: Beesley PL, Ross GJB, Glasby CJ, editors. Polychaetes and Allies: the Southern Synthesis. Melbourne: CSIRO Publishing; 2000. pp. 138–141.

117. Glasby CJ, Miura T, Nishi E, Junardi. A new species of *Namalycastis* (Polychaeta: Nereididae: Namanereidinae) from the shores of South-east Asia. Beagle. 2007;23: 21–27.

118. Paxton H. Revision of the genus *Micronereis* (Polychaeta: Nereididae: Notophycinae). Rec Aust Mus. 1983;35(1): 1–18. doi: 10.3853/j.0067-1975.35.1983.299.

119. Berkeley E, Berkeley C. *Micronereis nanaimoensis* sp. n.: with some notes on its life-history. J Fish Res Bd Can. 1953;10: 85–95. doi: 10.1139/f53-007.

120. Rullier F. Recherchés sur la morphologie et la reproduction du Nereidien *Micronereis variegata* Claparède. Arch Zool Exp Gen. 1954;91: 195–233.

121. Westheide W. A hermaphroditic *Sphaerosyllis* (Polychaeta: Syllidae) with epitokous genital chaetae from intertidal sands of the Island of Phuket (Thailand). Can J Zool. 1990;68: 2360–2363. doi: 10.1139/z90-327.

122. Kuper M, Westheide W. Ultrastructure of the male reproductive organs in the interstitial annelid *Sphaerosyllis hermaphrodita* ("Polychaeta", Syllidae). Zoomorphology. 1997;117: 13–22. doi: 10.1007/s004350050025.

123. Ehlers E. Neuseeländische Anneliden. Abh Konigl Ges Wiss Gottingen, MathPhys Kl, Neue Fol. 1904;3: 1–80.

124. Hutchings PA, Reid A, Wilson RS. *Perinereis* (Polychaeta, Nereididae) from Australia, with redescriptions of six additional species. Rec Aust Mus. 1991;43: 241–274. doi: 10.3853/j.0067-1975.43.1991.47.

125. Conde-Vela VM. Revision of *Laeonereis* Hartman, 1945 (Annelida: Phyllodocida: Gymnonereidinae), with a review of shaft morphology in nereidids. J Nat Hist. 2021;55(7–8): 381–455. doi: 10.1080/00222933.2021.1903601.

126. Conde-Vela VM. New species of *Pseudonereis* Kinberg, 1865 (Polychaeta: Nereididae) from the Atlantic Ocean, and a review of paragnath morphology and methodology. Zootaxa. 2018; 4471: 245–278. doi: 10.11646/zootaxa.4471.2.2.

127. Villalobos-Guerrero TF, Idris I. Redescriptions of a neglected species of *Pseudonereis* Kinberg, 1865 (Annelida: Nereididae) and its similar congener from the Eastern Tropical Pacific. J Nat Hist. 2020;54(23–24): 1559–1580. doi: 10.1080/00222933.2020.1810800.

128. Imajima M. Review of the annelid worms of the family Nereidae of Japan, with descriptions of five new species or subspecies. Bull Natn Sci Mus, Tokyo. 1972;15: 37–153.

129. Khlebovich VV, Komendantov AY, Shklyarevich GA. Validity of *Nereis virens, N. grandis* and *N. brandti* (Annelida, Polychaeta) and variations in the number of their paragnaths. Zool Zhurnal. 1980;59: 1617–1624. [in Russian].

130. Fitzhugh K. Phylogenetic relationships within the Nereididae (Polychaeta): implications at the subfamily level. Bull Biol Soc Wash. 1987;7: 174–183.

131. Hong JS, Choi BM, Kubo A, Sato M. Redescription of the giant mud worm *Paraleonnates uschakovi* Khlebovich and Wu, 1962 (Polychaeta: Nereididae) with special reference to the synonymy of *Periserrula leucophryna* Paik, 1977 and the difference from *Paraleonnates bolus* (Hutchings and Reid, 1991). Zootaxa. 2012;3490: 49–62. doi: 10.11646/zootaxa.3490.1.4.

132. Banse K. Gymnonereidinae new subfamily: the Nereididae (Polychaeta) with bifid parapodial neurocirri. J Nat Hist. 1977;11: 609–628. doi: 10.1080/00222937700770541.

133. Hylleberg J, Nateewathana A. Polychaetes of Thailand. Nereididae (Part 2): *Ceratocephale* and *Gymnonereis,* with description of two new species and notes of the subfamily Gymnonereidinae. Phuket Mar Biol Cent Res Bull. 1988;49: 1–20.

134. Santos CSG, Pleijel F, Lana P, Rouse GW. Phylogenetic relationships within Nereididae (Annelida: Phyllodocida). Invertebr Syst. 2005;19: 557–576. doi: 10.1071/IS05001.

135. Alves PR, Halanych KM, Silva EP, Santos CSG. Nereididae (Annelida) phylogeny based on molecular data. Org Divers Evol. 2023. doi: 10.1007/s13127-023-00608-9

136. Bakken T, Wilson RS. Phylogeny of nereidids (Polychaeta, Nereididae) with paragnaths. Zool Scr. 2005;34: 507–547.

137. Misra A. Polychaete. In: Gosh AK, editor. Zoological Survey of India, Calcutta, Series 3. State Fauna Series, Fauna of West Bengal, part 10, Invertebrates; 1999. pp. 125–225.

138. Day JH. The polychaete fauna of South Africa. Part 1. The intertidal and estuarine Polychaeta of Natal and Mosambique. Ann Natal Mus. 1951;12: 1–67.

139. Day JH. A monograph on the Polychaeta of Southern Africa. Part 1. Errantia. Bull. Br Mus Nat Hist. 1967;6: 1–458.

140. Hsueh PW. Two new species of nereidids (Annelida, Polychaeta) from Taiwan. Zootaxa. 2019;4652(3): 544–556.

Supporting information

S1 Table Accession numbers for the phylogenetic analysis of COI, 16S rRNA and 18S rRNA shown in S1 Fig. Data was sourced from Alves et al. 2023 [131]. New sequences are indicated in **bold.** Most sequence IDs from BOLD are marked with #. * the name *Laeonereis* cf. *pandoensis* (Monro, 1938) is used here instead of *Laeonereis culveri* (Webster, 1879) since the specimen was collected in Brazil. One terminal from Alves et al. 2023 [131] was not included here, *Ceratonereis longiceratophora* Hartmann-Schröder, 1985 as the there was no 16S sequence, the COI sequence (AY583701) is actually a flabelligerid and the 18S sequence appears to be of a hesionid. The correct spelling for sequences lodged on GenBank as *Tylorrhynchus heterochaetus* is *Tylorrhynchus heterochetus.*

S1 Fig. Maximum likelihood (ML) tree derived from analysis of nuclear 18S rRNA and mitochondrial COI and 16S rRNA DNA sequences. Support values at nodes are bootstrap support percentages after 1,000 pseudoreplicates.

S1 Video. Epitokous living specimens of *Pectinereis strickrotti* gen. nov., sp. nov. recorded at the type locality, swimming near the bottom at the Mound 12 methane seep at ~1,000m depth (8.930° N; 84.312° W) on Nov. 2, 2018 (dive 4987 of the DSV *Alvin).*

S2 Video. A solitary epitoke living specimen of *Pectinereis strickrotti* gen. nov., sp. nov., swimming at the Parrita Scar methane seep (8.951° N; 84.634° W) on January 11, 2019, at ~1,100 m recorded via ROV *SuBastian* on dive S0218.

Figures

Click here to access/download;Figure;Figures.pdf

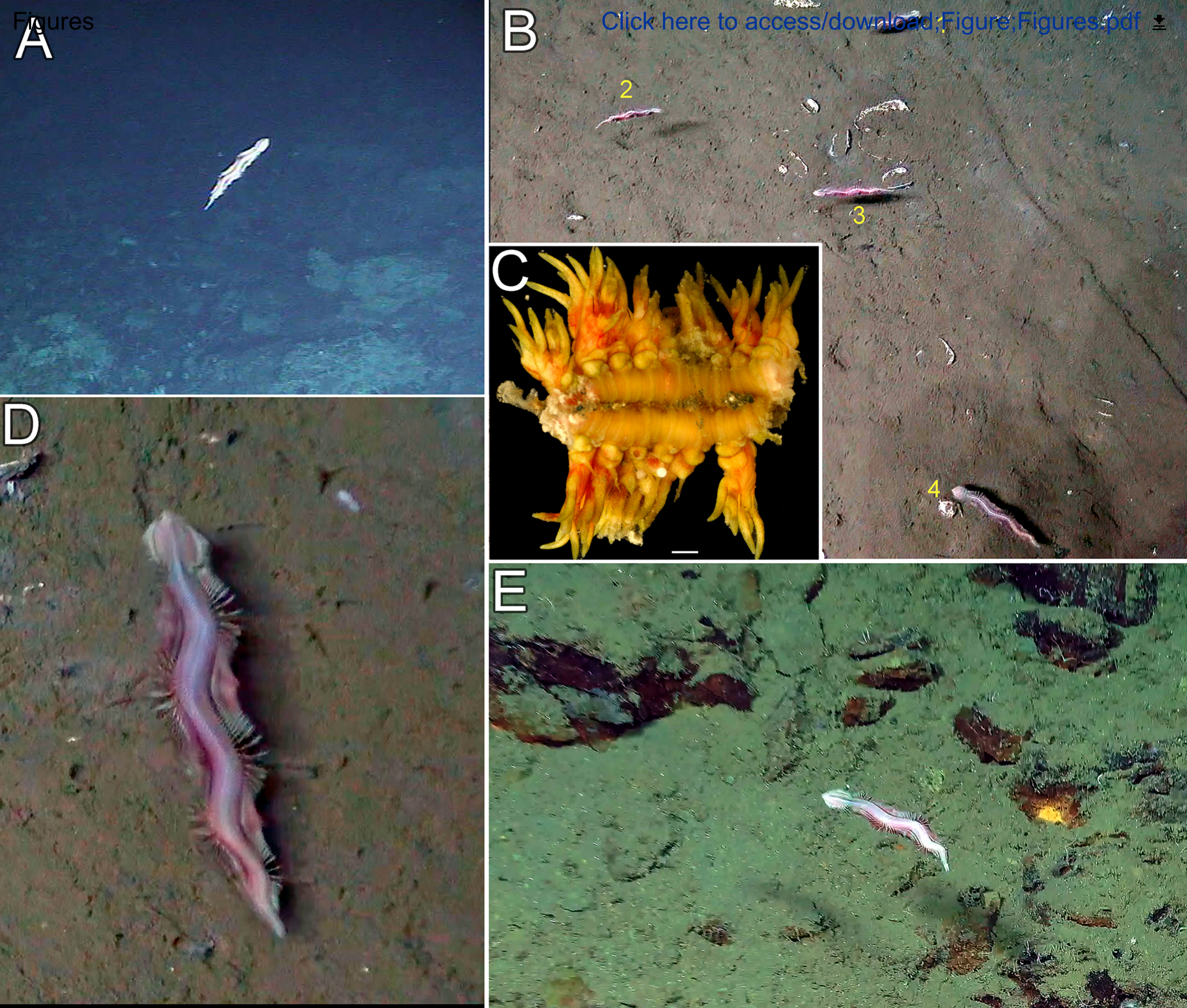

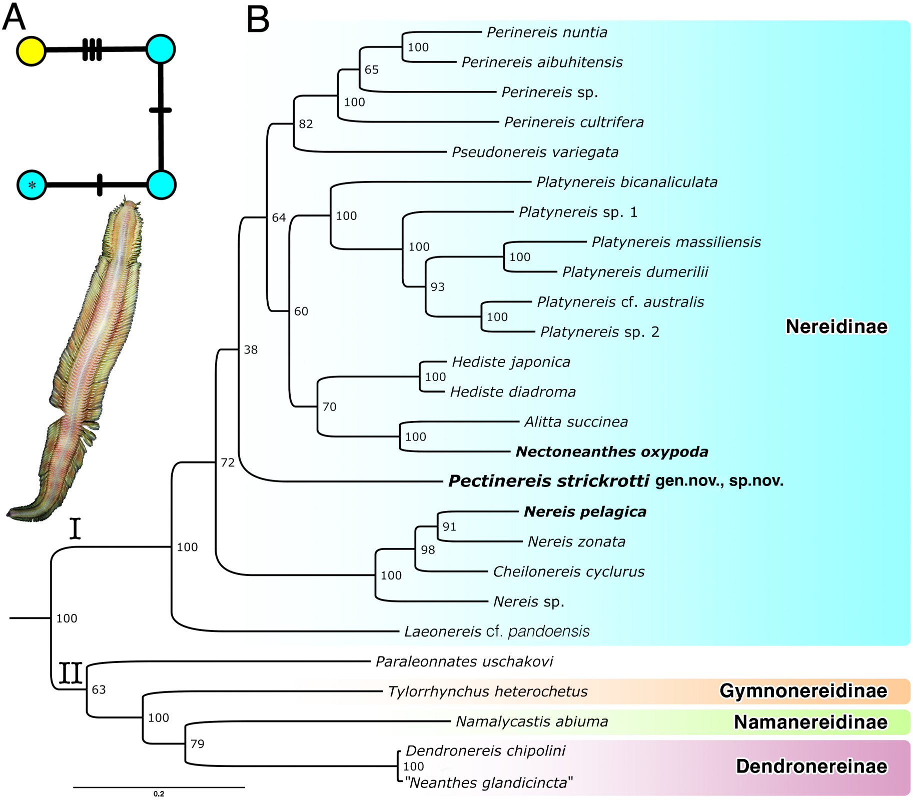
A
B
Perinereis nuntia
Perinereis aibuhitensis
Perinereis sp.
Perinereis cultrifera
Pseudonereis variegata
Platynereis bicanaliculata
Platynereis sp. 1
Platynereis massiliensis
Platynereis dumerilii
Platynereis cf. australis
Platynereis sp. 2
Hediste japonica
Hediste diadroma
Alitta succinea
Nectoneanthes oxypoda
Pectinereis strickrotti gen.nov., sp.nov.
Nereis pelagica
Nereis zonata
Cheilonereis cyclurus
Nereis sp.
Laeonereis cf. pandoensis
Paraleonnates uschakovi
Tylorrhynchus heterochetus
Namalycastis abiuma
Dendronereis chipolini
"Neanthes glandicincta"
Nereidinae
Gymnonereidinae
Namanereidinae
Dendronereinae
I
II
0.2

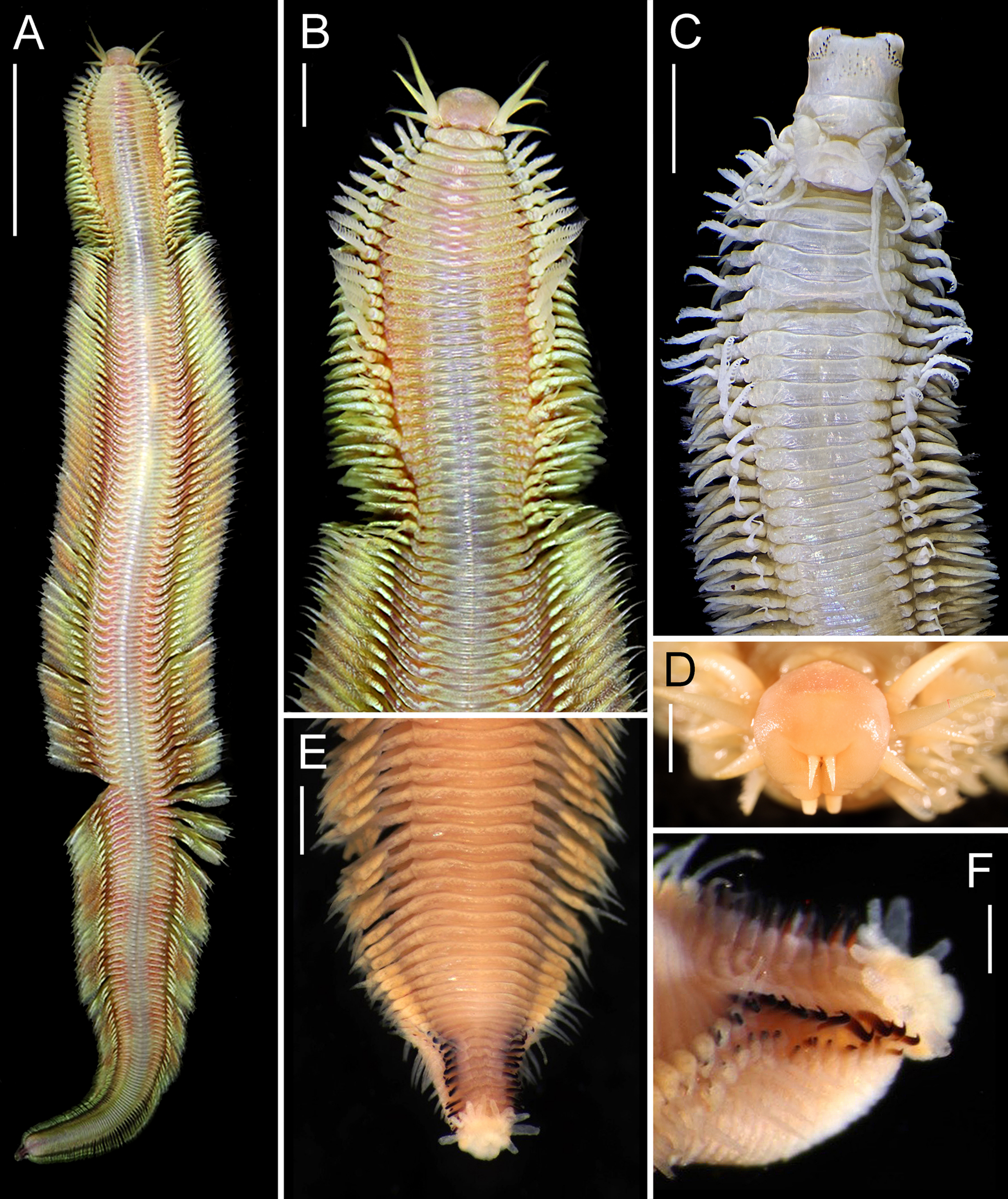
A
B
C
D
E
F

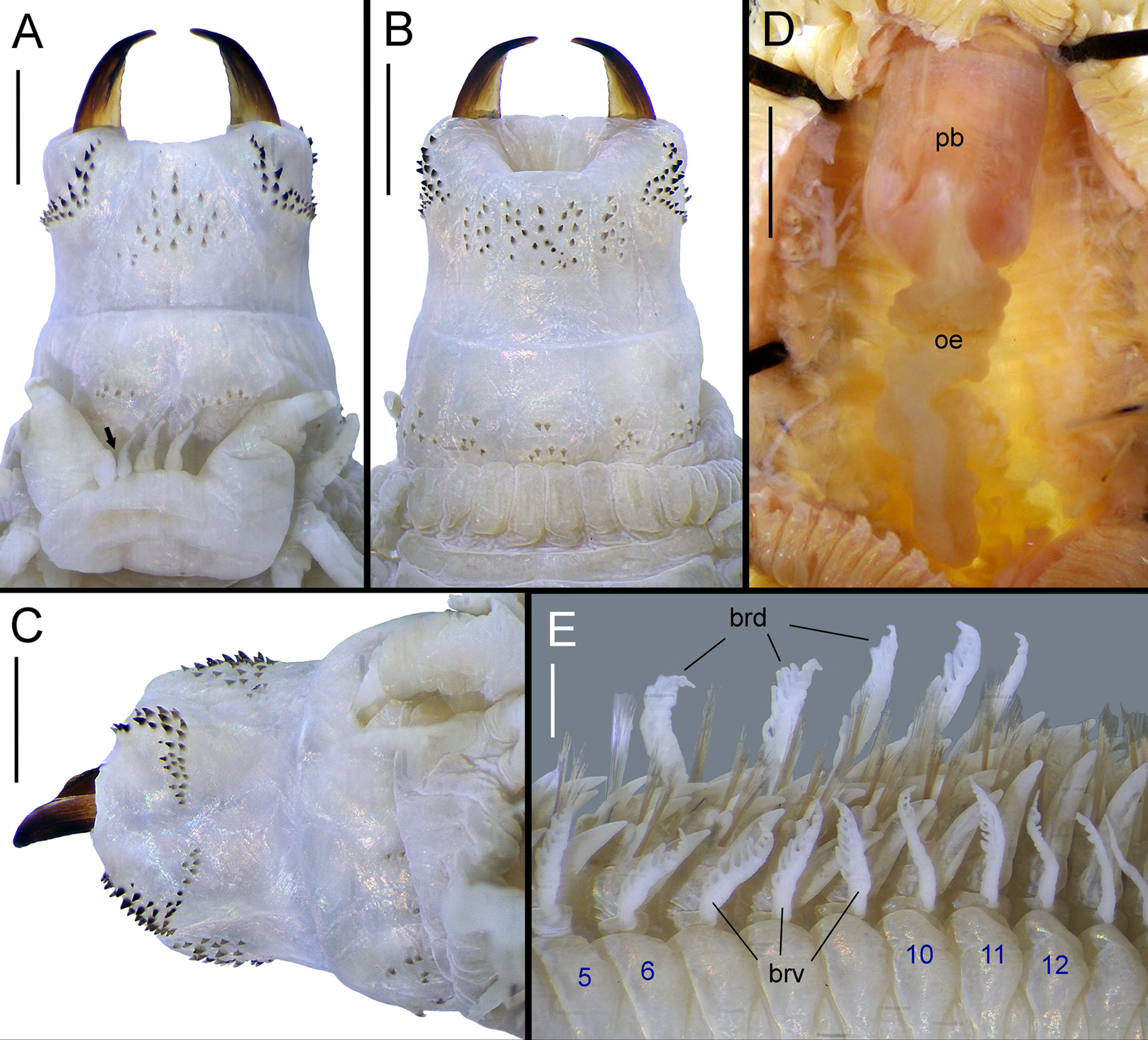
A
B
D
pb
oe
C
E
brd
brv
5
6
10
11
12

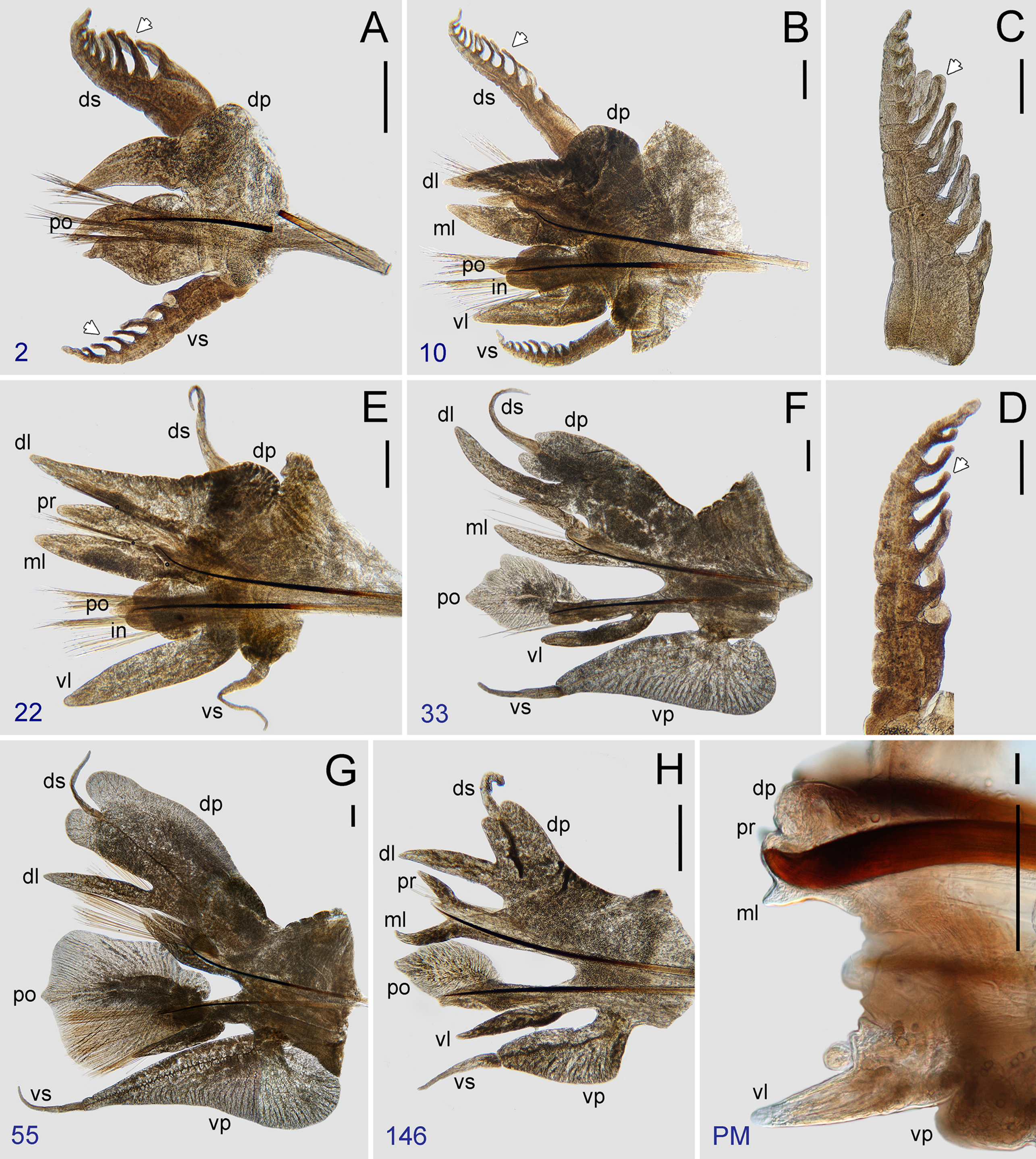

A
ds
dp
po
vs
2
B
ds
dp
dl
ml
po
in
vl
vs
10
C
E
dl
ds
dp
pr
ml
po
in
vl
vs
22
F
dl
ds
dp
ml
po
vl
vs
vp
33
D
G
ds
dp
dl
po
vs
vp
55
H
ds
dp
dl
pr
ml
po
vl
vs
vp
146
I
dp
pr
ml
vl
vp
PM

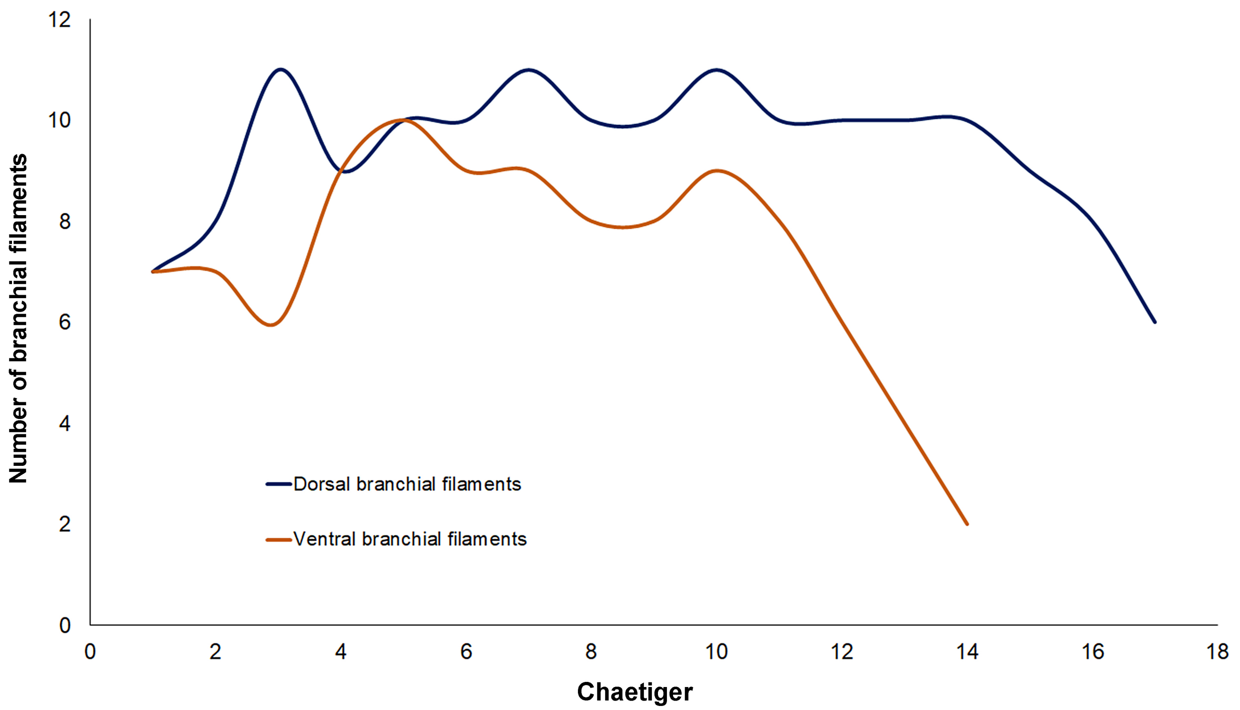

Number of branchial filaments
12
10
8
6
4
2
0
0
2
4
6
8
10
12
14
16
18
Chaetiger
Dorsal branchial filaments
Ventral branchial filaments

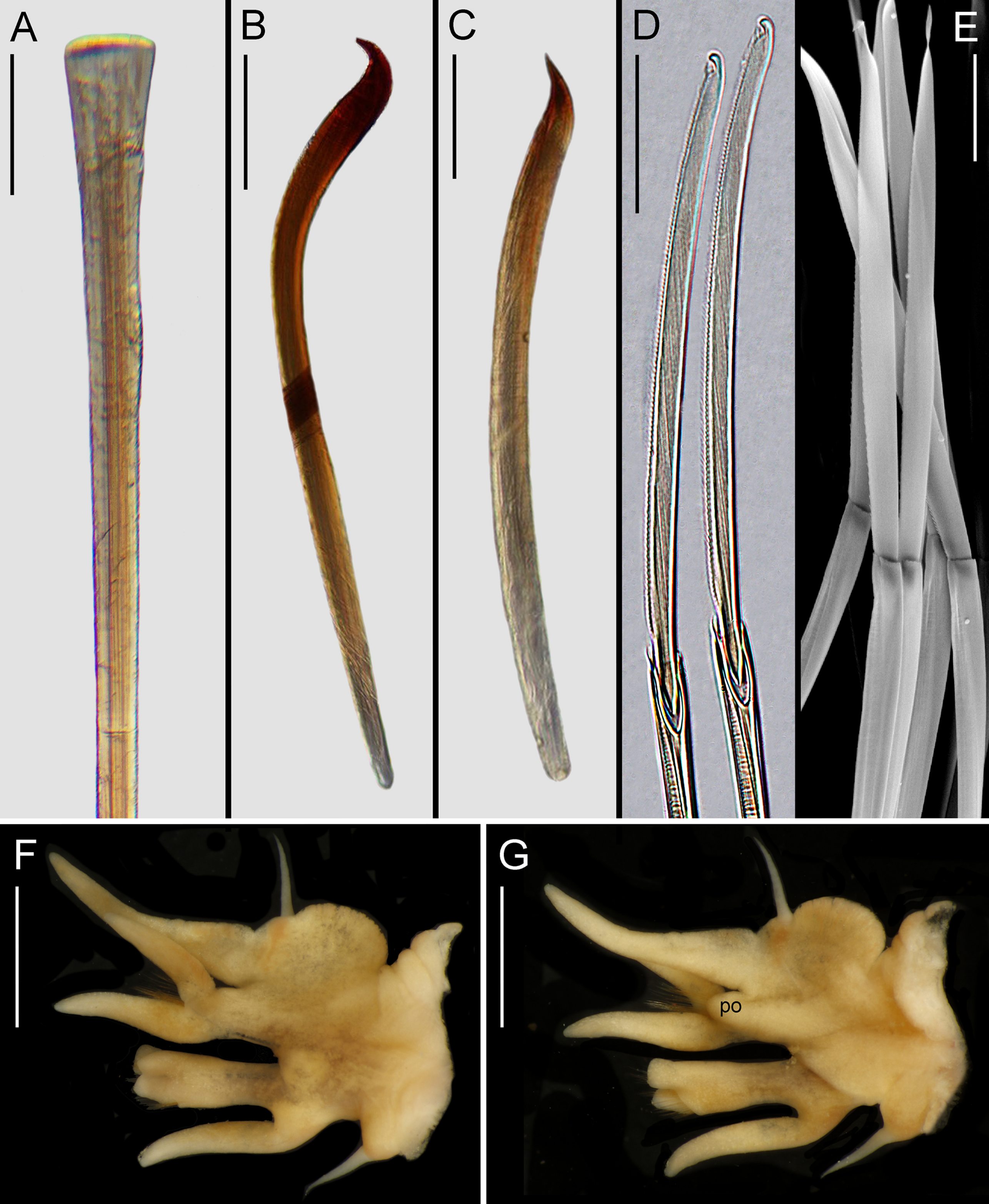

A
B
C
D
E
F
G
po

S1 Table Supporting Information

Click here to access/download
Supporting Information
S1Table.docx

S1 Figure

Click here to access/download
Supporting Information
S1_3Genetre.jpg

Data Review URL

Click here to download Data Review URL

http://www.dropbox.com/scl/fo/4rltnxu05nr0znhe8uanj/h?rlkey=9dnkdhoa
